# Threshold and mediating effects of new urbanization on residential building carbon emissions: evidence from Chinese pilot cities

**DOI:** 10.3389/fpubh.2025.1628610

**Published:** 2025-09-03

**Authors:** Yongkun Wang, Zhuo Tong, Yunhui Zhang

**Affiliations:** College of Business Administration, Liaoning Technical University, Huludao, China

**Keywords:** new urbanization, residential carbon emissions, environmental regulation, scientific and technological innovation, mediating effect, threshold effect, heterogeneity

## Abstract

**Introduction:**

In the context of China’s new urbanization strategy and its “dual-carbon” goals, understanding the impact of urban transformation on carbon emissions in the residential sector is crucial. This study explores the influence mechanism of new urbanization on carbon emissions from residential buildings using panel data from 58 pilot cities between 2012 and 2021.

**Methods:**

A comprehensive analytical framework incorporating fixed-effects, mediation, and threshold models is employed to examine the direct, mediating, and nonlinear effects of new urbanization.

**Results:**

The empirical findings indicate that: (1) new urbanization has a significant positive impact on residential building carbon emissions; (2) environmental regulation and scientific and technological innovation (STI) serve as mediators, each exhibiting emission-reducing effects within this relationship; (3) both environmental regulation and STI demonstrate double-threshold effects, with the regulatory impact diminishing at higher intensities, while the mediating effect of STI follows a nonlinear U-shaped trend; and (4) the mediating roles of environmental regulation and STI are subject to substantial regional and urban-size heterogeneity, being more effective in eastern regions and megacities.

**Discussion:**

These results offer new empirical insights into the carbon implications of urban development and provide policy guidance for differentiated, region-specific, and innovation-driven carbon reduction strategies in the residential sector.

## 1 Introduction

Environmental challenges driven by climate change have attracted increasing global attention. According to the Intergovernmental Panel on Climate Change (IPCC) Sixth Assessment Report, anthropogenic activities have contributed to a rise in global temperatures of approximately 0.8–1.2 °C since pre-industrial times. If current warming trends persist, global temperatures are projected to increase by 1.5 °C between 2030 and 2052 (1). To mitigate this trajectory, achieving near-zero carbon emissions in the energy sector by 2050 has become imperative. In response, China announced its commitment in September 2020 to peak carbon emissions by 2030 and achieve carbon neutrality by 2060 (2). Simultaneously, a strategic framework for new urbanization was introduced, emphasizing people-centered development as a vital pathway toward ecological civilization (3).

However, rapid urban population growth has led to a surge in carbon emissions, which presents significant challenges to high-quality urban development, undermines energy conservation efforts, and complicates the attainment of dual-carbon objectives. Among China’s major carbon-emitting sectors—industry, construction, and transportation—over 90% of total energy consumption and emissions are concentrated. Notably, the construction sector alone accounts for approximately one-third of the country’s energy consumption, with residential buildings contributing around 40% of that total, making them a primary source of emissions (4, 5). Although urbanization and increasing population density have driven the expansion of the construction industry, they have also intensified energy use and carbon dioxide emissions. In this context, the new urbanization paradigm emphasizes ecological progress, aiming to improve residents’ quality of life while reducing emissions from residential buildings. This underscores the complex and intertwined relationship between urbanization and carbon emissions.

Understanding this relationship has become a focal point of academic inquiry. Existing literature identifies three primary perspectives. First, some studies report no significant correlation between urbanization and carbon emissions (6, 7). Second, other studies suggest a linear relationship, though the direction remains contested. While some scholars argue that urbanization exacerbates emissions based on national panel data (8, 9), others find evidence of a mitigating effect, as shown by Huo et al. (10) and Wang et al. (11) using national and provincial datasets. The third school of thought proposes a nonlinear relationship. For example, studies have identified inverted “U” (12, 13) and “N” shaped curves across various regions of China (14). Multidimensional approaches, such as those by Feng et al. (15) and Hu et al. (16), emphasize that ecological, economic, and social factors may collectively mediate the influence of urbanization on emissions. In addition, sector-specific investigations provide further insights. Zheng et al. (17) demonstrate an inverted “U” relationship between urbanization and emissions in the digital economy, while Wang et al. (18) highlight spatial spillover effects from tourism. Lin et al. (19) examine emissions trends in the construction sector at the provincial level.

Despite these contributions, important gaps remain. Many studies adopt single-dimensional frameworks, lacking a comprehensive analysis of the multifaceted interactions between urbanization and carbon emissions. Moreover, much of the existing literature relies on provincial or national data, often overlooking city-level heterogeneity and spatial dynamics. Sector-specific impacts, particularly within the construction domain, are also underexplored, despite their significance to overall emissions.

To overcome the dual limitations prevalent in existing research - namely, the constrained single-dimensional analytical frameworks and the inadequate consideration of urban heterogeneity characteristics and spatial–temporal dynamics (20), the present study investigates 58 municipal pilot cities from 2012 to 2021. To more accurately quantify the level of new urbanization, this study first constructs a comprehensive evaluation system encompassing five key dimensions: population, economy, society, space, and ecology. To this end, the entropy weight method is employed to objectively weight indicators across these dimensions. Meanwhile, utilizing provincial carbon emission data and nighttime light data, city-level residential building carbon emissions are estimated through an inverse calculation approach. Furthermore, by establishing fixed-effects and mediation models, the study systematically analyzes the intrinsic mechanisms through which new urbanization influences residential building carbon emissions, thereby providing new empirical evidence for formulating differentiated low-carbon policies.

## 2 Theoretical analysis methods and research hypothesis

### 2.1 Influence mechanism

New urbanization represents a comprehensive and integrated development strategy that encompasses demographic, economic, social, spatial, and ecological dimensions. It aims to foster coordinated, efficient, low-carbon, and people-centered urban growth (3). From a theoretical standpoint, the impact of new urbanization on carbon emissions from residential buildings can be analyzed through three primary pathways: (1) a direct effect, (2) an indirect effect through mediating variables, and (3) a nonlinear effect characterized by threshold phenomena. The subsections below elaborate on these mechanisms and present the corresponding research hypotheses.

### 2.2 Direct effect

New urbanization influences carbon emissions from residential buildings through five primary mechanisms: (1) Population growth in urban areas intensifies the demand for household appliances, lighting, cooking equipment, and heating and cooling systems, leading to elevated carbon emissions. Concurrently, rising income levels, improved living standards, an increasing number of households, and shrinking household sizes all contribute to greater per capita energy consumption (21). (2) The growth in per capita GDP has resulted in higher wages and disposable incomes, driving up household energy consumption and associated carbon emissions. (3) Enhanced public services and social security systems attract more people to urban areas. For instance, broader pension coverage incentivizes rural-to-urban migration, thereby increasing residential energy demand and carbon output (22). (4) Urban Spatial Expansion: The influx of urban populations necessitates the development of expanded infrastructure—such as wider roads, new public buildings, and high-density residential complexes—which increases energy use both during and after construction (23). (5) Although ecological development is a core tenet of new urbanization, and greening efforts (e.g., increasing urban vegetation) can help sequester carbon and mitigate the urban heat island effect, these benefits are typically long-term (3). In contrast, the short-term impact of increased infrastructure investment may result in sustained energy demand and higher emissions (24). Consequently, hypothesis 1 suggests that new urbanization exerts a direct and positive effect on carbon emissions from residential buildings due to increased energy consumption associated with demographic, economic, and spatial development.

### 2.3 Indirect effect

New urbanization also exerts indirect effects on residential building carbon emissions through the promotion of environmental regulation and scientific and technological innovation (STI).

The advancement of new urbanization has significantly strengthened environmental regulatory frameworks, which serve as crucial instruments for reducing carbon intensity in the residential sector (25). Traditional urbanization processes often generated substantial environmental degradation, prompting the implementation of more stringent regulatory mechanisms in the new urbanization era. These include: pollution taxes and emissions trading systems, government subsidies for environmental protection, standardization of carbon emission metrics, incentives for energy-efficient appliances and green products. Such policy tools stimulate both producers and consumers to adopt sustainable practices, improve energy efficiency, and reduce household carbon emissions (26). Based on the above analysis hypothesis 2 is proposed: the new urbanization indirectly reduces residential building carbon emissions by enhancing environmental regulation, which acts as a mediating variable in this relationship.

STI is a key driver of social advancement and plays a vital role in achieving high-quality urban development (27). New urbanization emphasizes human capital development, technological progress, and the transition from primary and secondary industries toward tertiary sectors. This transition facilitates STI through: increased investment in research and development, adoption of low-carbon technologies and energy-efficient systems, and industrial structure optimization and cleaner energy integration. These technological innovations contribute to lowering residential energy demand and emissions through improved efficiency, smart building designs, and cleaner energy usage (28–30). Hypothesis 3: New urbanization indirectly reduces residential building carbon emissions by promoting scientific and technological innovation, which functions as a mediating variable in this process.

### 2.4 Threshold effect

The above analysis reveals that while new urbanization directly increases carbon emissions, environmental regulation and STI can mitigate this effect. However, the strength of these mediating effects may not be constant across all levels of development. This raises two key research questions (12–14): (1) Does the nonlinear relationship between new urbanization and carbon emissions—well-documented in aggregate analyses—also manifest in the residential building sector? (2) Is there an optimal threshold of environmental regulation or STI intensity beyond which their mediating effects are maximized? Understanding these threshold effects is critical for formulating effective policies, as it allows for the identification of inflection points where regulatory or technological investments yield the highest carbon mitigation benefits.

**Figure 1:**
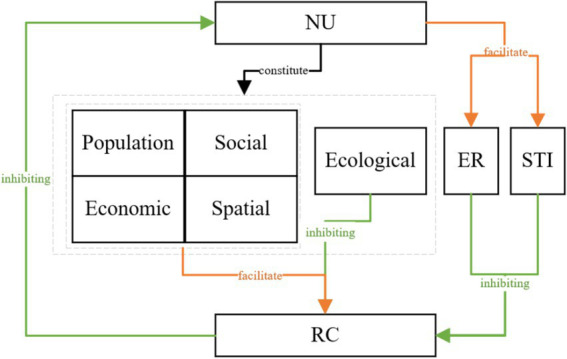
Conduction mechanism diagram.

Combining the above analyses, the transmission mechanism by which new urbanization affects carbon emissions from residential buildings is illustrated in Figure 1. Based on this insight, Hypothesis 4 is proposed: The impact of new urbanization on carbon emissions from residential buildings exhibits a nonlinear relationship, with environmental regulation and scientific and technological innovation acting as threshold variables, each possessing distinct optimal levels of effectiveness.

## 3 Research design

### 3.1 Research methodology and model setting

#### 3.1.1 Indicator measurement methodology

To accurately measure each sub-dimension index of new urbanization, this paper employs the entropy weight method. This method is chosen due to its high accuracy, broad applicability, strong objectivity, and superior ability to reflect changes in weights over time. The specific steps for applying the entropy weight method are based on the findings of Shang et al. (31).

#### 3.1.2 Carbon emission measurement methodology for residential buildings

As one of the most widely used methods for evaluating carbon emissions from energy consumption, the Emission Factor Method is recommended by the IPCC in its Guidelines for National Greenhouse Gas Inventories for calculating carbon emissions. This method involves multiplying the consumption of each carbon source by its corresponding carbon emission factor and then summing these values to estimate the total emissions for a region. The principle behind this approach is encapsulated in the following Equation 1:


(1) $${\mathrm{RC}}_{t}=\sum_{i=1}^{n}e_{\mathrm{it}}f_{i}$$

Where ${\mathrm{RC}}_{t}$ refers to the total carbon emissions from residential buildings in year t, $e_{\mathrm{it}}$ refers to the consumption of energy i in year t, while $f_{i}$ corresponds to the carbon emission factor for energy i and n represents the type of energy.

#### 3.1.3 Econometric modeling

Through the above analysis, considering that there are many factors affecting carbon emissions from residential buildings, it is challenging to include all their influencing factors in the regression model, and the endogeneity issues arising from the omitted variables can be effectively dealt with in the fixed-effects model, so the baseline regression model of this study is constructed as follows in Equation 2:


(2) $$\ln RC_{\mathrm{it}}=a_{0}+a_{1}NU_{\mathrm{it}}+a_{c}X_{\mathrm{it}}+u_{i}+\delta_{t}+\varepsilon_{\mathrm{it}}$$

where $RC_{\mathrm{it}}$ is the carbon emissions from residential buildings in year t, $NU_{\mathrm{it}}$ is the level of new urbanization development in city i in year t, $X_{\mathrm{it}}$ is a series of control variables, $a_{0}$ denotes the intercept term, $a_{1}$ denotes the estimated parameters of the core explanatory variables, $u_{i}$ represents the city fixed effects, $\delta_{t}$ represents the year fixed effects, and $\varepsilon_{\mathrm{it}}$ is the random error term.

In order to test the transmission mechanism of new urbanization on residential carbon emissions, i.e., whether environmental regulation and scientific and technological progress affect the relationship between the two, and to test whether there is a mediating effect between the two. Based on Hayes’s study (32) using the Bootstrap method with higher statistical validity, the model is constructed as follows:


(3) $$M_{\mathrm{it}}=\beta_{0}+\beta_{1}NU_{\mathrm{it}}+\beta_{c}X_{\mathrm{it}}+u_{i}+\delta_{t}+\varepsilon_{\mathrm{it}}$$

(4) $$\ln RC_{\mathrm{it}}=\gamma_{0}+\gamma_{1}NU_{\mathrm{it}}+\gamma_{2}M_{\mathrm{it}}+\gamma_{c}X_{\mathrm{it}}+u_{i}+\delta_{t}+\varepsilon_{\mathrm{it}}$$

Where M is the mediator variable Equation 3 is the regression model of NU on the mediator variable M and Equation 4 is the regression model of NU and the mediator variable M on RC . The presence of the mediating effect was determined based on whether the 95% confidence interval contained zero.

In order to test the nonlinear effect of the impact of new urbanization on carbon emissions from residential buildings and the stage effect of the mediating variables, the threshold effect model proposed by Hansen is used for further analysis. And drawing on Lian’s (33) treatment, the threshold variables are double-tested. The following model is established:


(5) $$\begin{array}{l} \ln RC_{\mathrm{it}}=\omega_{0}+\omega_{1}NU_{\mathrm{it}}\times I(M\leq q_{1})+\omega_{2}NU_{\mathrm{it}}\times I(q_{2}\leq M\leq q_{2})+\\ \omega_{3}NU_{\mathrm{it}}\times I(M>q_{2})+\text{ωcXit}+\mathrm{\delta t}+\mathrm{\varepsilon it} \end{array}$$

where M denotes the threshold variable, $q_{i}$ denotes the threshold value, I is the indicator function, and the rest is the same as the above equation.

### 3.2 Variable selection

#### 3.2.1 Explanatory variables

This paper selects the level of new urbanization (NU) as the explanatory variable. New urbanization goes beyond mere population growth; it represents a multifaceted phenomenon that must be assessed from various perspectives. To evaluate the level of new urbanization effectively, it is essential to integrate the people-centered urban development strategy with contemporary development concepts (16), ensuring a true reflection of the quality of new urbanization. Informed by the National New Urbanization Plan (2014–2020), the National New Urbanization Plan (2021–2035), and previous scholarly methods (34, 35), the evaluation system categorizes new urbanization into five sub-dimensions: demographic, economic, social, spatial, and ecological. Initially, considered 50 indicators for evaluation, but some were excluded due to insufficient relevance or suitability at the city level. After Using Ge ‘s approach (36), covariance and the coefficient of variation were applied to refine the indicators, resulting in the retention of eighteen. Detailed results are presented in Table 1.

**Table 1:** Indicator system for the development level of new urbanization.

Index	Index weights	Index II	Unit	Index attribute	Index II weights
Population	0.0972	Population density	Person/km^2^	+	0.017
		Percentage of population	%	+	0.0397
		Unemployment rate	%	−	0.0405
Economy	0.2436	Tertiary industry/GDP	%	+	0.0563
		Per capita GDP	10^4^Yuan	+	0.0718
		Per capita disposable Income	Yuan	+	0.0831
		Per capita salary	Yuan	+	0.0324
Social	0.264	Old-age insurance rate	%	+	0.0743
		Per capita Road area	M^2^	+	0.0796
		Library collection/10^2^	Album	+	0.0877
		Number of doctors/10^4^	Person	+	0.0224
Spatial	0.3085	Road network density	Km/km^2^	+	0.1681
		Floor area	Km^2^	+	0.0827
		Completed/Urban area	%	+	0.0577
Ecological	0.0867	Domestic garbage disposal	%	+	0.013
		Green coverage rate	%	+	0.0185
		Per capita green space	M^2^	+	0.0428
		Sewage treatment rate	%	+	0.0124

#### 3.2.2 Dependent variables

**Figure 2:**
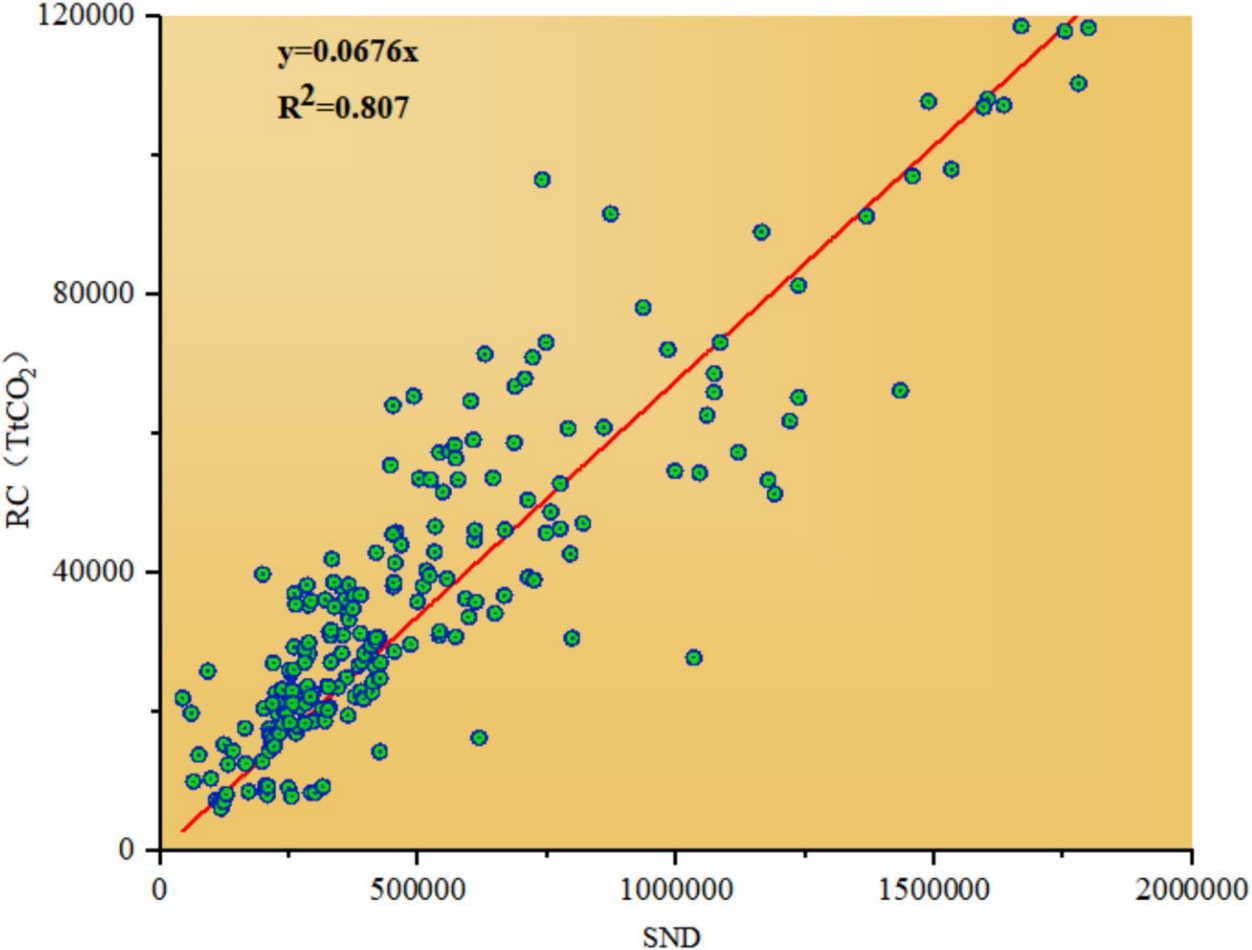
Fitting curve of provincial total nighttime light value and carbon emissions from residential buildings.

This paper selects residential building carbon emissions (RC) as the explanatory variable. Due to the absence of comprehensive energy statistics at the municipal level, calculating municipal carbon emissions directly by using the emission factor method is not feasible. However, scholars have validated the reliability of estimating CO^2^ emissions through inverse extrapolation from nighttime lighting data (37, 38). Based on this approach, this study employs ArcGIS to extract the total value of nighttime lighting (SDN) in each province, then linearly fits this data to carbon emissions calculated by the emission factor method. Considering the accuracy issues in the process of model dimensionality reduction and inversion, this study adopts a linear model without an intercept term to describe the linear relationship between the total provincial nighttime light value (SDN) and the carbon emissions of residential buildings, with λ denoting the parameters to be estimated. Thus, the formula RC=λ×SDN is obtained. The fitting results are illustrated in Figure 2.

#### 3.2.3 Mediating variables

Drawing on the research of previous scholars (30, 39), this paper selects environmental regulation (ER) as one of the mediating variables, and uses the investment in urban appearance and environmental sanitation to reflect the intensity of environmental regulation. The rationale is that environmental regulation involves three primary stakeholders: government, enterprises, and residents. Investment in amenities and sanitation signals the high priority governments place on such regulations, with greater investment typically indicating more stringent regulatory policies and measures. This encourages enterprises to adopt green technologies to minimize pollution emissions. Simultaneously, enhanced amenities improve residents’ quality of life and bolster their environmental awareness, fostering broader policy support and cooperation.

Another crucial variable, STI (40, 41), is measured by the number of granted invention patents. Invention patents are a vital indicator of technological innovation, manifesting originality and technological breakthroughs. They can effectively reflect the level of STI in a region or field, providing a quantifiable basis for relevant research and analysis. A high number of granted patents indicates robust R&D activities, effective translation of research into practical applications, and a strong emphasis on intellectual property within a city. This metric not only reflects the city’s innovative vitality and technological advancement but also highlights solid industrial support and a conducive innovation environment.

#### 3.2.4 Control variables

To address potential omissions that might affect the equity of urban residential carbon emissions, several control variables are included to provide a comprehensive analysis. The industrial structure (IS) (39) optimizes the industrial environment, limits the expansion of high-polluting industries, and facilitates industrial upgrades that directly reduce carbon emissions. Regions with higher economic levels (EL) (30) typically experience accelerated urbanization, leading to increased building energy consumption and carbon emissions. Economic activities with high energy intensity (EI) (40) require more energy to sustain, generally resulting in heightened carbon emissions. An increase in floor area (FA) (41) raises demand for heating, cooling, electricity, and materials, thus elevating energy use and carbon emissions. Government capacity (GC) (42) influences policy development, the promotion of energy efficiency standards, and the application of green technologies, all aimed at minimizing building-related carbon emissions. These factors are measured as follows: the proportion of secondary and tertiary industries (IS); the logarithm of fixed asset investment (EL); energy consumption per unit of GDP (EI); the land area used for urban residential buildings in the current year (FA); and the sum of public finance revenue and expenditure (GC).

### 3.3 Data sources and descriptive statistics

The data for indicators and control variables used to evaluate the level of new urbanization were sourced from the 2012–2022 editions of the China Urban Statistical Yearbook, China Urban and Rural Construction Statistical Yearbook, and the Statistical Bulletin of National Economic and Social Development of Cities, as well as the Statistical Yearbook of Cities. Information relevant to calculating carbon emissions from residential buildings was extracted from the 2012–2022 editions of the China Energy Statistical Yearbook, the China Urban and Rural Construction Statistical Yearbook, and the 2006 Guidelines for National Greenhouse Gas Inventories. Descriptive statistics of the collected data are presented in Table 2.

**Table 2:** Descriptive statistics for variables.

Variable	*N*	Mean	SD	Min	Max
NU	580	6.566	1.473	3.540	10.178
RC	580	0.275	0.076	0.123	0.607
IS	580	1.107	0.455	0.051	4.053
EL	580	7.697	0.982	2.887	10.657
EI	580	0.064	0.042	0.008	0.252
FA	580	3.848	0.917	0.542	6.322
GC	580	15.725	0.922	13.058	18.639
ER	580	5.478	9.227	0.005	105.655
STI	580	6.627	1.669	1.792	10.453

## 4 Empirical results and analysis

### 4.1 Analysis of new urbanization levels in pilot cities

**Figure 3:**
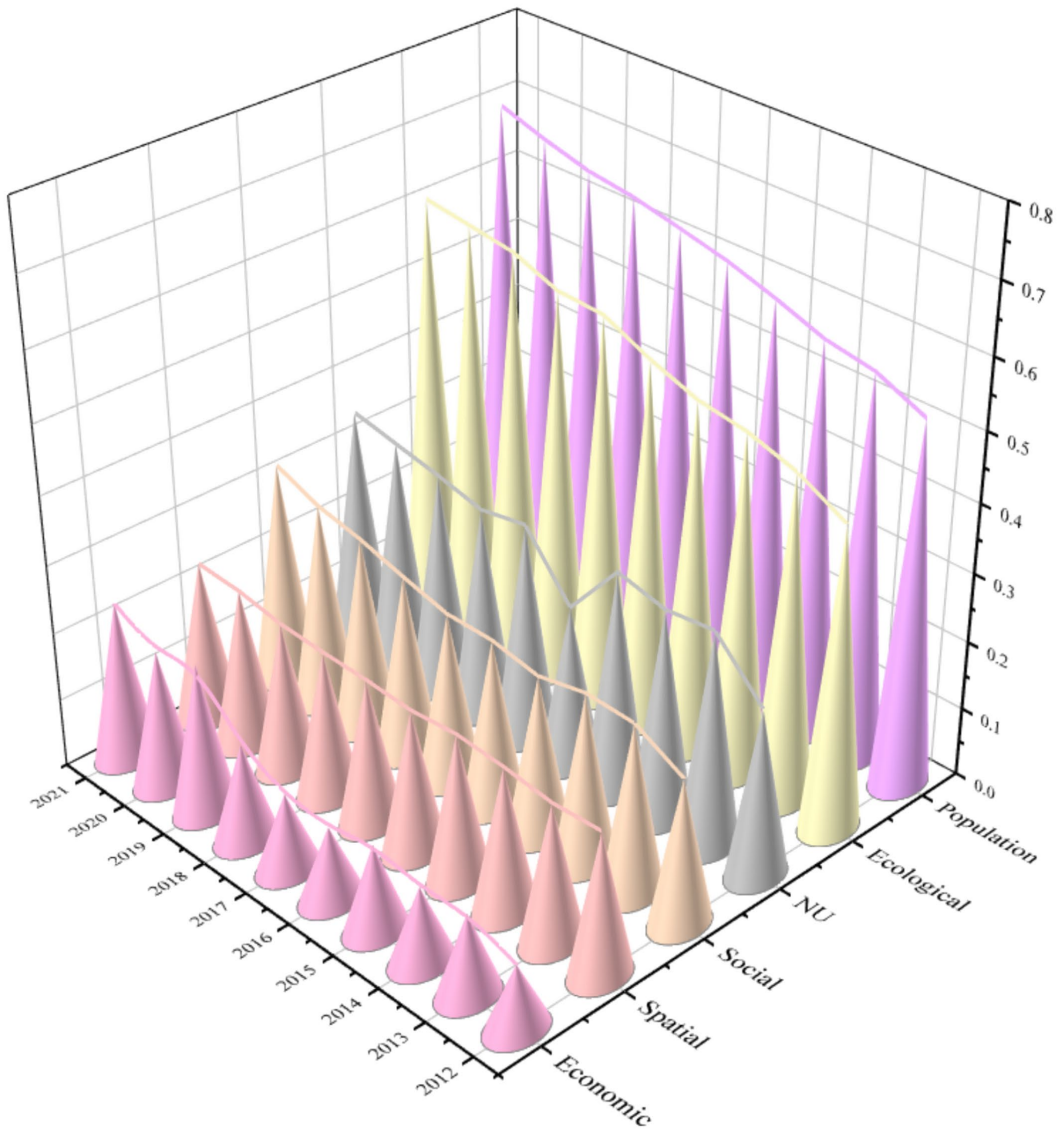
Trends in indicator scores.

The entropy weight method was employed to evaluate the level of new urbanization across multiple dimensions, and the results are visually presented in Figure 3. The figure illustrates a steady upward trend in urbanization development scores, reflecting comprehensive progress across all dimensions. Among these, population urbanization achieved the highest score, indicating a pronounced concentration of population in urban areas, followed by ecological urbanization. Although social and spatial urbanization exhibited relatively lower scores, both dimensions demonstrated gradual improvement over time.

In terms of growth rates, the overall level of new urbanization increased by 25.82% during the study period. Notably, economic urbanization experienced the most substantial growth, rising by 138.79%, which underscores the pivotal role of economic restructuring as the main driver of new urbanization. Social urbanization followed, while demographic and ecological urbanization showed comparable growth trajectories. In contrast, spatial urbanization advanced at a slower pace, with a growth rate of only 11.89%. When considering average annual growth, economic urbanization stood out with a rate of 10.09%, highlighting its strong momentum and underlying economic dynamism.

Furthermore, substantial regional disparities were observed among the pilot cities in terms of their urbanization development levels. The Eastern region consistently outperformed both the Central and Western regions, with the Central region demonstrating moderate but steady progress. At the city level, eastern cities such as Nanjing, Suzhou, and Ningbo ranked among the highest in terms of new urbanization, whereas western cities like Luzhou, Qujing, and Anshun remained at the lower end of the spectrum. This regional heterogeneity can be attributed to differences in industrialization, economic strength, infrastructure quality, and technological capacity. The Eastern region benefits from advanced industrial bases, strong economic performance, and superior infrastructure—all of which are critical to driving new urbanization. In contrast, the Western region faces structural challenges, including complex topography and underdeveloped transportation networks, which constrain economic development and limit urban expansion.

Understanding these regional disparities is essential for identifying the fundamental causes of uneven development and for formulating targeted policy interventions. Such efforts can promote more balanced, inclusive, and coordinated urbanization across different regions of China.

### 4.2 Analysis of carbon emissions from residential buildings in pilot cities

**Figure 4:**
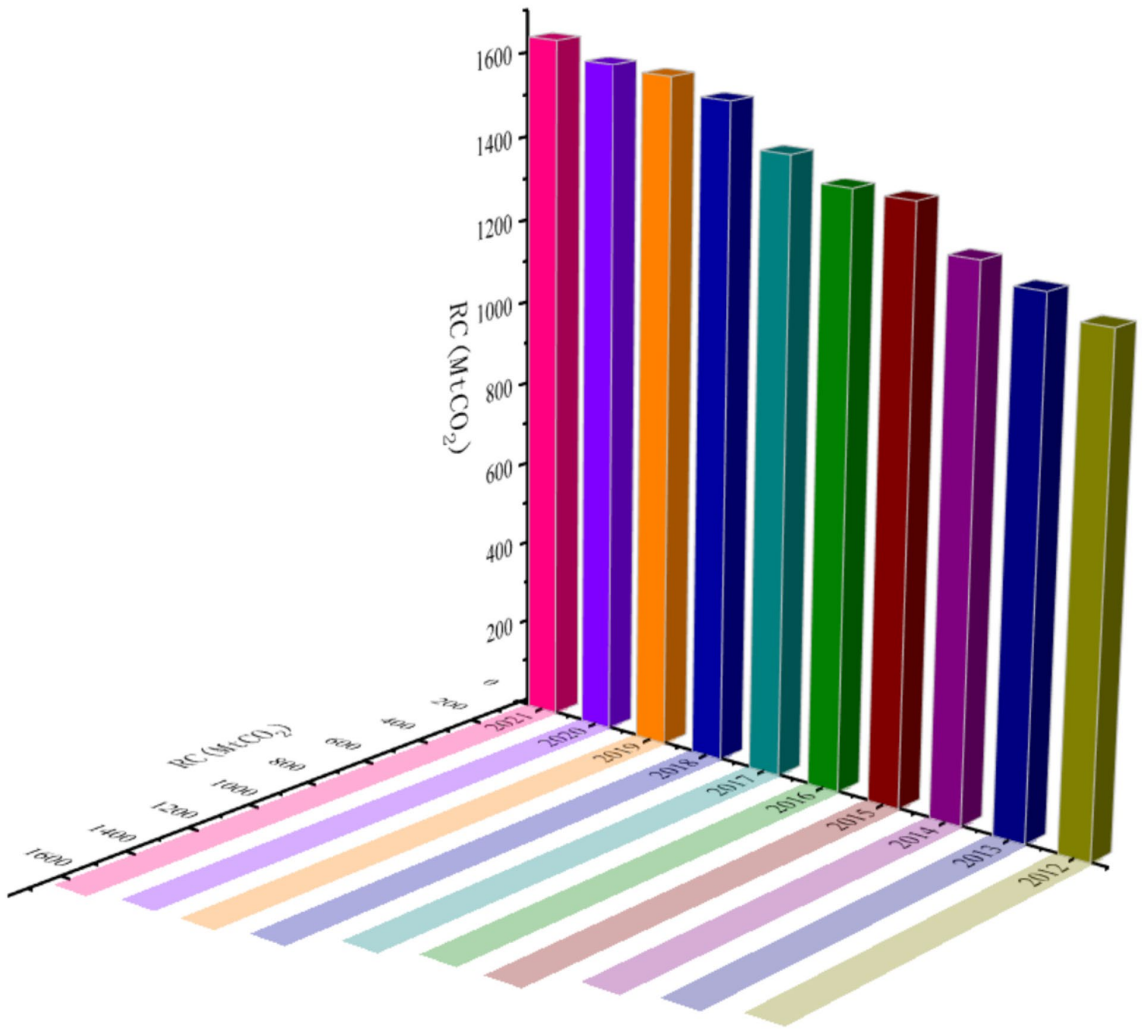
Trends in carbon emissions from residential buildings.

Carbon emissions from residential buildings at the city level were estimated using nighttime light data in conjunction with provincial residential carbon emission figures. Based on this estimation, the temporal and spatial trends of carbon emissions in the pilot cities were analyzed, as illustrated in Figure 4. Overall, the period from 2012 to 2021 witnessed a continuous rise in residential building carbon emissions across these cities. Notably, the rate of increase slowed during the latter half of the study period (2016–2021) compared to the earlier years (2012–2015), suggesting that new urbanization initiatives have contributed to curbing the acceleration of emissions growth.

A spatial analysis of the data reveals a clear regional pattern: carbon emissions from residential buildings generally increase from west to east, with a pronounced concentration in the northeastern region. This spatial distribution underscores significant regional heterogeneity. In western cities, lower carbon emissions are primarily due to lower population densities and the relatively delayed onset of urbanization, both of which contribute to reduced building energy consumption. In contrast, eastern cities are characterized by dense commercial and residential development, leading to greater reliance on energy-intensive systems such as air conditioning, lighting, and household appliances, thereby resulting in higher carbon emissions.

Recognizing and understanding these regional disparities is essential for formulating targeted strategies to mitigate carbon emissions. Tailored approaches that account for the distinct urbanization patterns and energy use behaviors in different regions will be critical to achieving sustainable, low-carbon urban development.

### 4.3 Direct effect test analysis

To begin the empirical analysis, panel unit root tests were conducted for all variables in the dataset. The results indicate that all variables, or their first-order lag terms, are stationary, thereby satisfying the requirements for subsequent panel data analysis. Following this, both correlation coefficient and variance inflation factor (VIF) tests were performed to assess potential multicollinearity among the independent variables. As shown in Table 3, the absolute values of all pairwise correlation coefficients are below 0.8, and the VIF values for all variables are less than 10. These findings confirm the absence of multicollinearity, ensuring the reliability of the regression results.

**Table 3:** Covariance and correlation test.

Variable	VIF	lnRC	NU	IS	EL	EI	FA	GC	ER	STI
lnRC		1								
NU	1.36	0.577	1							
IS	2.51	−0.411	−0.081	1						
EL	4.13	0.479	0.246	−0.306	1					
EI	1.82	0.630	0.019	−0.384	0.398	1				
FA	3.62	0.540	0.098	−0.360	0.607	0.508	1			
GC	7.58	0.509	0.218	−0.440	0.601	0.536	0.740	1		
ER	1.83	−0.248	0.217	−0.291	0.215	−0.494	0.233	0.300	1	
STI	2.88	−0.343	0.201	−0.556	0.170	−0.160	0.062	0.021	0.362	1

Subsequently, both fixed-effects and random-effects models were applied to conduct benchmark regressions. The Hausman test was employed to determine the most appropriate model specification. The test results supported the fixed-effects model, indicating that it yields more consistent and efficient estimates.

To further account for both individual (city-level) and time (yearly) effects, regressions were performed using individual fixed-effects and two-way fixed-effects models. Columns (1) and (2) in Table 4 present the baseline regression results for new urbanization (NU) on residential building carbon emissions (lnRC). In both models, NU is positively and significantly associated with carbon emissions at the 1% significance level, confirming Hypothesis 1: that new urbanization contributes to an increase in residential building carbon emissions.

**Table 4:** Benchmark regression results.

Model	(1)	(2)	(3)	(4)	(5)
Variable	lnRC	lnRC	lnRC	lnRC	lnRC
NU	7.5737^***^	8.1371^***^	8.4418^***^	8.0221^***^	9.1046^***^
	(0.8392)	(0.2465)	(0.3208)	(0.1106)	(0.2386)
IS			−0.0436	0.1349^***^	−0.0955^***^
			(0.0378)	(0.0157)	(0.0346)
EL			0.4102^***^	0.3268^***^	0.4133^***^
			(0.0314)	(0.0114)	(0.0272)
EI			16.6429^***^	8.8658^***^	15.5653^***^
			(0.4347)	(0.2701)	(0.4249)
FA			0.1161^***^	0.1012^***^	0.0760^**^
			(0.0417)	(0.0145)	(0.0321)
GC			0.1731^***^	0.0234	0.0915^**^
			(0.0405)	(0.0150)	(0.0364)
_cons	4.8599^***^	4.1065^***^	−3.0356^***^	−1.2311^***^	−1.6278^***^
	(0.1970)	(0.0628)	(0.6323)	(0.2444)	(0.4763)
Year	No	Yes	No	Yes	No
Ind	Yes	Yes	Yes	Yes	No
*N*	580	580	580	580	580
adj. *R*^2^	0.3326	0.9184	0.8657	0.9844	0.8757

***, **, and * represent 1, 5, and 10% levels of significance.

Columns (3) and (4) extend the baseline analysis by incorporating control variables—industrial structure (IS), economic level (EL), energy intensity (EI), floor area (FA), and government capacity (GC). The positive and highly significant coefficient for NU remains robust, reaffirming the initial finding.

Among the control variables, IS, EL, EI, and FA are all positively and significantly associated with carbon emissions at the 1% level. Notably, EI exhibits the largest effect size, underscoring the critical role of energy efficiency in emission reduction efforts. The coefficient for GC is positive but statistically insignificant in the two-way fixed-effects model.

The significant positive coefficient for IS suggests that a higher proportion of secondary industry activity hinders efforts to reduce residential building emissions. The positive impact of EL reflects a dual mechanism: economically developed cities tend to prioritize growth over ecological sustainability in early urbanization phases, while their increased population density and commercial activity further elevate residential energy consumption. EI, as a measure of energy efficiency, indicates that higher energy intensity—implying lower efficiency—leads to greater energy use for basic activities such as heating and electricity, thereby increasing emissions. Similarly, FA contributes positively and significantly to emissions, as expanded building areas require more energy for lighting, heating, and cooling.

Comparing model fits, the two-way fixed-effects model (columns 2 and 4) achieves higher adjusted R^2^ values than the individual fixed-effects model, suggesting that controlling for both city and year effects provides better explanatory power. The random-effects model presented in column (5), while still significant, shows a lower R^2^ value than the two-way fixed-effects model, corroborating the Hausman test results and justifying the selection of the two-way fixed-effects model for subsequent analyses.

### 4.4 Analysis of the mediating effects test

The preceding analysis confirms that new urbanization significantly increases carbon emissions from residential buildings. To further examine the mechanisms underlying this relationship—specifically Hypotheses 2 and 3—Equation 3 is employed to test the mediating effects of environmental regulation and scientific and technological innovation (STI). The regression results are presented in Table 5.

**Table 5:** Mediation effect regression and test results.

Model		(1)	(2)	(3)	(4)	(5)
Variable		lnRC	ER	lnRC	STI	lnRC
NU		8.0221***	2.8729**	8.0230***	1.9383**	8.0613***
		(0.1106)	(1.4443)	(0.1107)	(0.8676)	(0.1101)
ER				−0.0003***		
				(0.0001)		
STI						−0.0202***
						(0.0060)
_cons		−1.2311***	1.1820***	0.2308***	18.7226***	0.6092**
		(0.2444)	(0.4134)	(0.0826)	(1.7795)	(0.2667)
Sobel			−0.338***		0.276***	
Mediated			3.51%		2.86%	
Bootstrap	Indirect		[−0.4719, −0.2137]		[0.1662, 0.3898]	
	Direct		[9.5916, 10.4640]		[9.0323, 9.7626]	
CVs, Year, Ind		Yes	Yes	Yes	Yes	Yes
*N*		580	580	580	580	580
adj. R2		0.9844	0.7961	0.9843	0.8378	0.9847

***, **, and * represent 1, 5, and 10% levels of significance.

Column (1) reports the baseline regression of new urbanization on residential carbon emissions, showing a significantly positive coefficient for new urbanization. Columns (2) and (4) examine the influence of new urbanization on environmental regulation and STI, respectively. Columns (3) and (5) then incorporate these mediating variables into the baseline regression to assess their impact on carbon emissions.

In Column (2), the coefficient for new urbanization is significantly positive at the 5% level, indicating that the development of new urbanization promotes the strengthening of environmental regulation. In Column (3), when environmental regulation is included as a mediating variable, its coefficient is significantly negative, while the coefficient for new urbanization remains significantly positive. This suggests that increased regulatory intensity contributes to lower residential carbon emissions, even as urbanization progresses. The opposite signs of the new urbanization coefficient in Column (2) and the environmental regulation coefficient in Column (3) imply a masking effect—environmental regulation partially offsets the positive effect of new urbanization on emissions. Specifically, this masking effect accounts for 0.11‰ of the total effect, confirming the partial mediating role of environmental regulation.

Similarly, Column (4) demonstrates a significantly positive association between new urbanization and STI at the 5% level, indicating that urbanization development fosters technological progress. In Column (5), where STI is introduced as a mediating variable, its coefficient is significantly negative at the 1% level, while new urbanization remains positively associated with carbon emissions. The opposing signs between the coefficient of new urbanization in Column (4) and the STI coefficient in Column (5) also indicate a masking effect, wherein STI mitigates the positive influence of urbanization on emissions. This masking effect accounts for 4.86‰ of the total effect, suggesting that technological innovation plays a more substantial mediating role than environmental regulation.

Taken together, these results validate Hypotheses 2 and 3, confirming that both environmental regulation and STI function as mediators that reduce the impact of new urbanization on carbon emissions from residential buildings. Although new urbanization has a stronger influence on environmental regulation than on STI, the latter exerts a more pronounced mediating effect in curbing emissions. Therefore, enhancing STI capacity may offer a more effective strategy for mitigating urbanization-driven carbon emissions in the residential sector.

### 4.5 Threshold effect test analysis

#### 4.5.1 Threshold effect test

Before constructing the panel threshold effects model, two essential tests were conducted to ensure the robustness and validity of the threshold estimations. The first is the threshold significance test, which evaluates whether the estimated parameter values differ significantly across subgroups delineated by potential threshold values. This test primarily utilizes the F-statistic and its associated *p*-value to determine statistical significance. The second is the threshold estimate accuracy test, which employs the Bootstrap method to assess the consistency and reliability of the threshold estimates. This method uses the likelihood ratio (LR) statistic to verify whether the identified thresholds align with the true values.

To examine Hypothesis 4, Equation 5 was applied, and the corresponding results are presented in Table 6. The findings reveal that the double-threshold effect of environmental regulation is statistically significant at the 1% level, confirming the presence of two thresholds. The F-statistic for the triple-threshold test, however, is not significant, indicating that the effect does not extend beyond two thresholds. Similarly, for STI, the single-threshold and double-threshold *F*-values are 11.07 and 10.62, respectively—both significant at the 5% level. This confirms the presence of a double-threshold effect for STI as well, while the triple-threshold result remains insignificant.

**Table 6:** Threshold effect test results.

Variable	Number	*F*	*p*	10%	5%	1%
ER	Single	57.34***	0.000	12.6239	14.1906	17.976
	Double	17.87***	0.000	7.5097	8.8174	11.9087
	Triple	10.13	0.510	17.3341	20.5861	24.3048
STI	Single	11.07**	0.050	9.7112	11.0156	13.2404
	Double	10.62**	0.023	9.0063	9.7543	11.9029
	Triple	5.09	0.873	23.9891	26.1418	30.9116

***, **, and * represent 1, 5, and 10% levels of significance.

**Figure 5:**
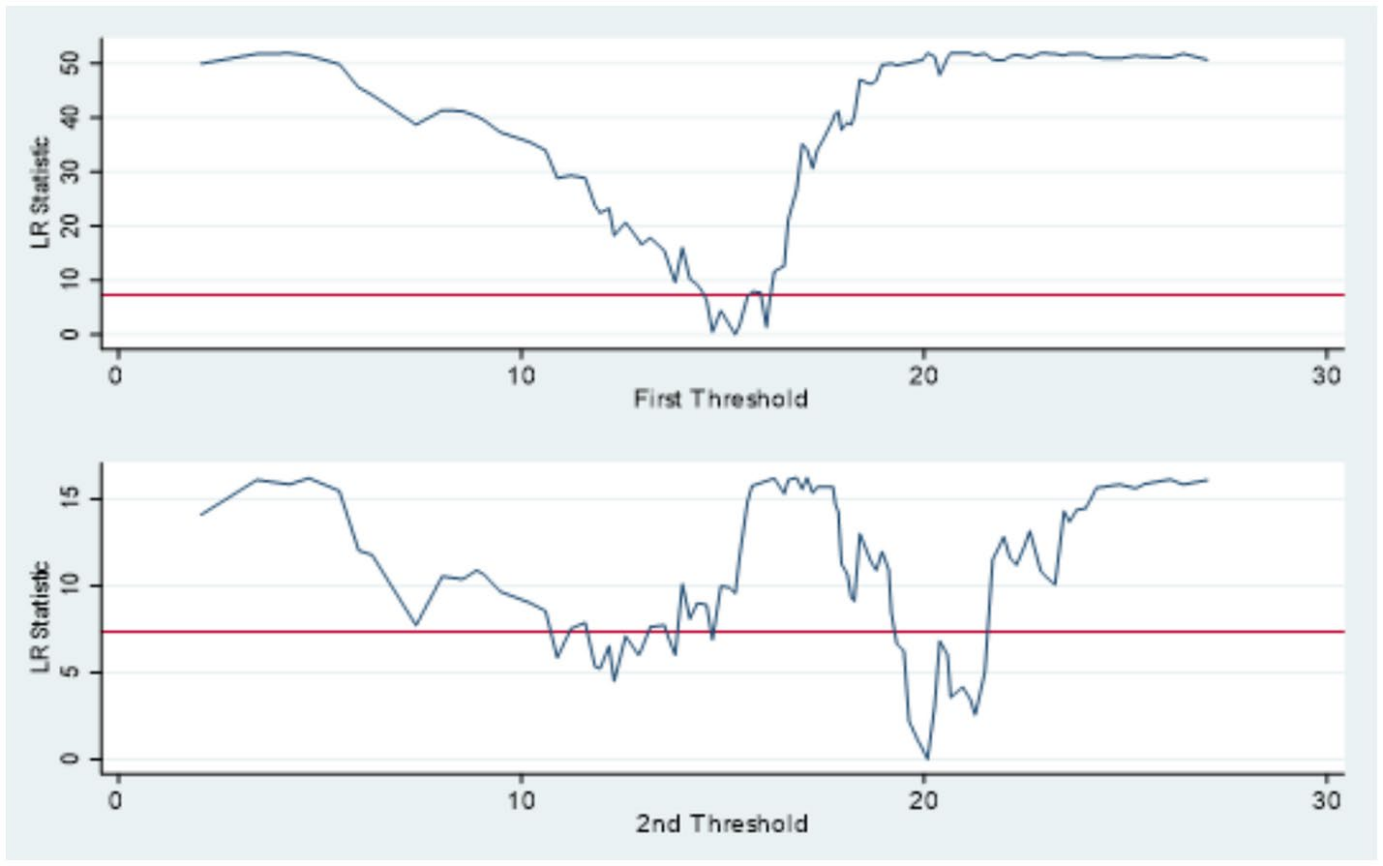
Double threshold for ER LR.

**Figure 6:**
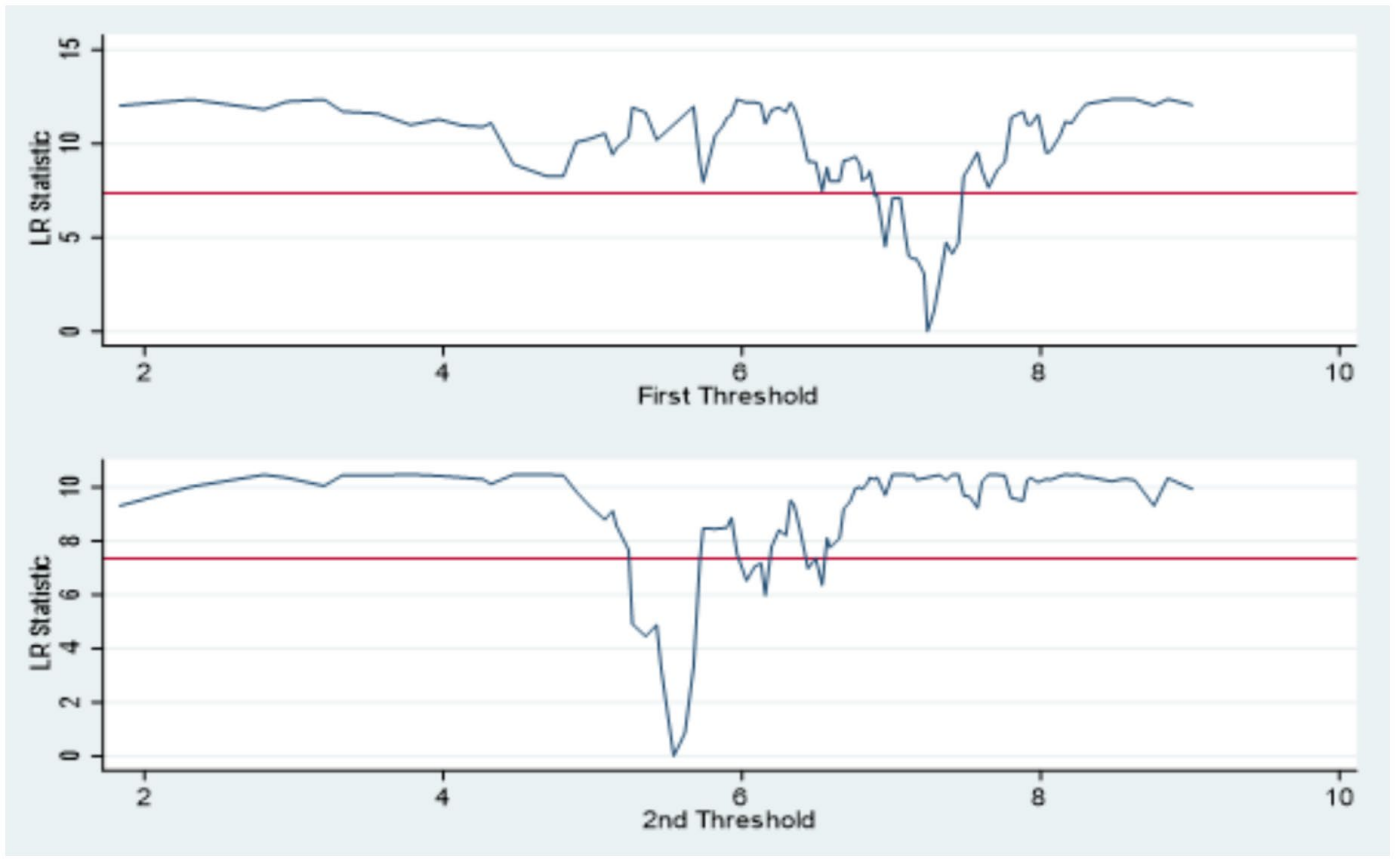
Double threshold for STI LR.

Further validation is provided in Table 7, which presents the estimated threshold values and their corresponding 95% confidence intervals. The identified thresholds for environmental regulation are 15.3112 and 20.0849, while those for STI are 5.5436 and 7.2422. These results are graphically supported by the likelihood ratio curves shown in Figures 5, 6, which affirm the stability and validity of the double thresholds through visual inspection of the LR statistics.

**Table 7:** Threshold effect test results.

Variable	Number	Value	95%confidence interval	
ER	Single	15.3112	14.8194	15.4233
	Double	20.0849	19.5705	20.2605
STI	Single	7.2422	7.0371	7.2848
	Double	5.5436	5.3501	5.6188

In summary, the analysis confirms the presence of a nonlinear relationship between new urbanization and carbon emissions from residential buildings. Both environmental regulation and STI demonstrate significant double-threshold effects, indicating that their mediating roles vary across different intensities. These threshold effects highlight the importance of optimal regulatory and technological development levels in mitigating the environmental impacts of urbanization.

#### 4.5.2 Analysis of threshold regression results

To further test the strength and effective range of the mediating effect under different thresholds, adopt the mediated threshold effect model, drawing on the approaches of Guo et al. (43) and Qin et al. (44) for deeper analysis.

Table 8 presents the mediation test results for environmental regulation (ER) across three threshold ranges. Columns (1)–(3) show results when ER is below the first threshold (ER ≤ 15.3112); columns (4)–(6) represent the intermediate range (15.3112 < ER ≤ 20.0849); and columns (7)–(9) reflect the upper range (ER > 20.0849).

**Table 8:** Regression results on the mediating threshold effect of ER.

	0 < ER ≤ 15.3112			15.3112 < ER ≤ 20.0849			ER > 20.0849			Model
(1)	(2)	(3)	(4)	(5)	(6)	(7)	(8)	(9)	Variable
lnRC	ER	lnRC	lnRC	ER	lnRC	lnRC	ER	lnRC
NU	7.4133^***^	20.8524^*^	7.9390^***^	9.2003^***^	6.1534^**^	9.5538^***^	8.1781^***^	−8.8150	8.0757^***^
	(1.0365)	(12.2884)	(1.0007)	(0.8454)	(2.8701)	(0.8436)	(0.6248)	(8.7443)	(0.6220)
ER			−0.0252^***^			−0.0574^**^			−0.0116^*^
			(0.0063)			(0.0256)			(0.0066)
cons	−0.7344	53.4448^***^	0.6128	−4.3831^**^	63.2142^***^	−0.7521	−1.9399^*^	34.7225^**^	−1.5364
	(1.2021)	(14.2513)	(1.1984)	(1.7661)	(6.0084)	(2.3745)	(1.1214)	(15.6930)	(1.1350)
Ratio	70.88‰			38.39‰			0		
CVs Year Ind	Yes	Yes	Yes	Yes	Yes	Yes	Yes	Yes	Yes
*N*	214	214	214	191	191	191	175	175	175
R^2^	0.7984	0.4580	0.8153	0.6419	0.3763	0.6528	0.7617	0.5638	0.764

***, **, and * represent 1, 5, and 10% levels of significance.

The results indicate that environmental regulation exhibits a significant mediating effect in both the lower and middle threshold intervals. Specifically, the mediating effect accounts for 70.88‰ of the total effect when ER is below the first threshold, and 38.39‰ when ER is between the first and second thresholds. However, when ER exceeds the second threshold, its mediating effect becomes statistically insignificant.

These findings confirm the presence of a double-threshold effect in environmental regulation, with thresholds identified at 15.3112 and 20.0849. In the first threshold range, new urbanization is positively and significantly associated with carbon emissions at the 1% level. It is also positively correlated with ER, while ER itself shows a significant negative effect on carbon emissions, thereby acting as a mediator. This suggests that, in the early phase of urban development, enhancing environmental regulation effectively suppresses the growth of residential building emissions.

In the middle threshold range, ER continues to mediate the impact of new urbanization, but its relative contribution decreases. This indicates diminishing marginal returns to the mediating role of ER as its intensity increases. Once ER surpasses the second threshold (ER > 20.0849), the correlation between ER and new urbanization becomes statistically insignificant, nullifying the mediating effect.

This pattern suggests that environmental regulation is most effective in the early to intermediate stages of urbanization. Initially, as regulatory frameworks strengthen, they play a vital role in controlling emissions. However, once regulatory efforts reach a certain saturation point, their marginal impact declines. This could be due to escalating economic and administrative costs associated with stricter regulations, such as higher compliance burdens, costlier pollution control measures, and stringent building codes. These factors may lead to noncompliance or regulatory evasion, thereby undermining the effectiveness of environmental regulation in mitigating carbon emissions.

Table 9 displays the mediation test results for scientific and technological innovation (STI) across three threshold intervals: columns (1)–(3) for STI ≤ 5.5436, columns (4)–(6) for 5.5436 < STI ≤ 7.2422, and columns (7)–(9) for STI > 7.2422.

**Table 9:** Regression results on the mediating threshold effect of STI.

	0 < STI ≤ 5.5436			5.5436 < STI ≤ 7.2422			STI > 7.2422			Model
(1)	(2)	(3)	(4)	(5)	(6)	(7)	(8)	(9)	Variable
lnRC	STI	lnRC	lnRC	STI	lnRC	lnRC	STI	lnRC
NU	9.3139^***^	7.4721^***^	9.4645^***^	9.0478^***^	1.8423^***^	9.0854^***^	7.8156^***^	2.2682^***^	8.0583^***^
	(0.2638)	(1.2395)	(0.2699)	(0.2093)	(0.5041)	(0.2101)	(0.5440)	(0.5332)	(0.1819)
STI			−0.0249^**^			−0.0275^*^			−0.0554^***^
			(0.0119)			(0.0163)			(0.2024)
_cons	−1.4237^***^	18.3720^***^	−1.0593^***^	−1.3258^***^	12.4874^***^	−0.9759^**^	−1.1601^***^	12.1707^***^	−0.1674
	(0.3396)	(1.9458)	(0.3806)	(0.3549)	(0.9657)	(0.4768)	(0.4125)	(1.3211)	(0.4609)
Mediated	19.98‰			5.60‰			41.14‰		
CVs Year Ind	Yes	Yes	Yes	Yes	Yes	Yes	Yes	Yes	Yes
*N*	149	149	149	239	239	239	192	192	192
*R* ^2^	0.9748	0.7628	0.9760	0.9818	0.7566	0.9844	0.8255	0.6895	0.9875

***, **, and * represent 1, 5, and 10% levels of significance.

Across all three intervals, STI consistently serves as a significant mediating factor in the relationship between new urbanization and residential carbon emissions. The masking effects, representing the proportion of the total effect mediated by STI, are 19.98‰, 5.60‰, and 41.14‰ respectively—indicating an initial decline followed by a notable increase in the mediating impact.

According to the double-threshold test results, STI exhibits significant thresholds at 5.5436 and 7.2422. Throughout all three stages, new urbanization is positively associated with both carbon emissions and STI at the 1% level, confirming that urbanization fosters technological advancement. At the same time, STI exerts a negative effect on carbon emissions, statistically significant at the 10% level or higher, and its inhibitory strength increases progressively across the threshold intervals.

This consistent masking effect suggests that STI plays a persistent role in attenuating the emissions impact of new urbanization. In the initial stage, STI helps reduce emissions by promoting energy-efficient building designs and technologies. However, as urbanization intensifies and population density rises, the surge in housing demand leads to escalating energy consumption, potentially outpacing the benefits of current STI capabilities. Additionally, certain advanced emission-reducing technologies may face financial, structural, or technological barriers that limit their large-scale implementation.

Nevertheless, in the third threshold range (STI > 7.2422), the mediating effect of STI strengthens significantly, accounting for 41.14‰ of the total effect. This indicates that as STI overcomes early-stage limitations—through technological breakthroughs, cost reductions, or broader policy support—it regains and enhances its capacity to mitigate carbon emissions from residential buildings.

### 4.6 Heterogeneity analysis

To explore the potential heterogeneous effects of environmental regulation and scientific and technological innovation (STI) on carbon emissions from residential buildings, the 58 pilot cities were further categorized based on regional and size-based criteria established by the National Bureau of Statistics of China. Two main classification rules were applied:

1. Geographic heterogeneity, which distinguishes between eastern and midwestern cities, and.
2. City size heterogeneity, which separates megacities (population ≥ 5 million) from small and medium-sized cities (S-A-M) (population < 5 million).

As shown in Table 10, the influence of environmental regulation on carbon emissions exhibits clear regional and size-based differences. In the eastern region, the coefficient for ER is −0.0296 and statistically significant, indicating a strong and effective inhibitory effect on emissions—likely due to better policy implementation, infrastructure, and regulatory enforcement.

**Table 10:** Results of the heterogeneity test for ER.

Model		(1)	(2)	(3)	(4)	Level
East		Midwest	Mega	S-A-M	Variable
lnRC		lnRC	lnRC	lnRC
NU		11.1940***	8.2192***	10.6953***	9.6595***
		(0.217)	(0.238)	(0.360)	(0.193)
ER		−0.0296***	−0.0139***	−0.0538***	−0.0157***
		(0.004)	(0.003)	(0.006)	(0.003)
_cons		−1.4098***	−1.1532**	4.6172***	1.7626***
		(0.184)	(0.557)	(1.274)	(0.493)
Sobel		−0.4633***	−0.2164	−0.5089***	−0.1939***
Mediated		4.317%	2.703%	4.995%	2.049%
Bootstrap	Indirect	[−0.6759, −0.2405]	[−0.4162, 0.1774]	[−1.1137, −0.1381]	[−0.3152, −0.0728]
	Direct	[10.8276, 11.6369]	[7.6807, 8.7681]	[9.9489, 11.4872]	[9.1977, 10.1213]
CVs/Yea/Ind		Yes	Yes	Yes	Yes
*N*		290	290	110	470
adj. R^2^		0.9444	0.9269	0.9277	0.9117

***, **, and * represent 1, 5, and 10% levels of significance.

In contrast, midwestern cities also show a significant negative ER coefficient (−0.0139), though smaller in magnitude. This suggests that while environmental regulation is functioning, its impact is more limited, possibly due to less developed infrastructure and weaker enforcement mechanisms.

Among city size categories, megacities demonstrate the strongest regulatory effect (ER coefficient = −0.0538), supported by concentrated resources, advanced technological infrastructure, and heightened environmental awareness. In comparison, S-A-M cities show a weaker but still significant effect (ER coefficient = −0.0157), potentially reflecting constraints in funding, administrative capacity, or policy implementation.

The Sobel test further supports these findings: the mediation effect of ER is most pronounced in eastern cities (4.317%) and megacities (4.995%), indicating that ER serves as a more effective mediating mechanism in these contexts. The bootstrap confidence intervals for indirect effects also confirm the statistical robustness of these mediating roles, especially in the eastern and megacity subsamples.

Similar patterns of heterogeneity are observed for STI, as presented in Table 11. In eastern cities, STI exhibits a strong dampening effect on carbon emissions (coefficient = −0.1126), suggesting the successful implementation of policies and technological measures. In midwestern cities, while the effect is somewhat smaller (coefficient = −0.0884), it remains statistically significant, indicating that STI still contributes to emissions mitigation despite regional development disparities.

**Table 11:** Results of the heterogeneity test for STI.

Model		(1)	(2)	(3)	(4)	Level
East		Midwest	Mega	S-A-M	Variable
lnRC		lnRC	lnRC	lnRC
NU		10.3458***	7.8844***	9.7289***	9.1727***
		(0.222)	(0.232)	(0.452)	(0.182)
STI		−0.1126***	−0.0884***	−0.1161***	−0.1091***
		(0.016)	(0.017)	(0.029)	(0.012)
_cons		−4.0065***	−2.6835***	−1.4208***	−3.6376***
		(0.637)	(0.645)	(1.453)	(0.533)
Sobel		0.3849***	0.1184	0.4576**	0.2929***
Mediated		3.587%	1.479%	4.499%	3.904%
Bootstrap	Indirect	[0.1837, 0.5860]	[−0.0211, 0.2379]	[0.0836, 0.8315]	[0.1617, 0.4241]
	Direct	[9.9003, 10.79126]	[7.3401, 8.4286]	[8.7144, 10.7433]	[8.7392, 9.6061]
CVs//Year/Ind		Yes	Yes	Yes	Yes
N		290	290	110	470
adj. R2		0.9417	0.9286	0.8906	0.9217

***, **, and * represent 1, 5, and 10% levels of significance.

Among city size categories, megacities display the most substantial reduction effect (STI coefficient = −0.1161), highlighting their greater capacity for deploying advanced technologies and strict emissions strategies. Meanwhile, S-A-M cities also demonstrate a significant STI effect (coefficient = −0.1091), suggesting that well-targeted policy support can yield meaningful carbon reductions even under more constrained conditions.

The Sobel test and associated mediation effect analyses reinforce the above observations. STI shows the highest mediation effect in megacities (4.499%), followed by eastern cities (3.587%) and S-A-M cities (3.904%). Although the midwestern region exhibits a smaller mediation proportion (1.479%), its positive direction suggests untapped potential. The bootstrap confidence intervals for indirect effects do not include zero for the eastern, megacity, and S-A-M categories, confirming statistical significance and robust mediation effects in these subgroups.

Importantly, all models report adjusted R^2^ values above 0.89, indicating excellent model fit across regional and city size categories.

These results underscore the heterogeneous effectiveness of environmental regulation and STI in reducing residential building carbon emissions. Both mediating mechanisms perform more effectively in eastern regions and megacities, likely due to better infrastructure, more advanced technological bases, and stronger governance capacity. In contrast, midwestern and smaller cities may require additional policy support, capacity building, and infrastructure investment to maximize the benefits of environmental and technological interventions. Accordingly, future emission reduction strategies should be tailored to local conditions, leveraging regional strengths while addressing area-specific constraints to advance balanced and sustainable urban development.

### 4.7 Robustness tests

To ensure the reliability of the empirical findings, a series of robustness tests were conducted, including variable substitution, the addition of control variables, exclusion of outliers, and restriction of the sample period. The results are summarized in Table 12.

**Table 12:** Robustness test results.

Model	(1)	(2)	(3)	(4)	Variable
InRC	InRC	InRC	InRC
NU	1.2718***	8.5614***	9.5835***	8.5749***
	(0.360)	(0.321)	(0.438)	(0.415)
IS	0.0609	−0.0609	−0.0276	0.0028
	(0.057)	(0.038)	(0.056)	(0.049)
EL	0.4488***	0.3988***	0.4509***	0.4643***
	(0.048)	(0.031)	(0.045)	(0.050)
EI	16.3369***	16.4445***	16.0400***	15.8414***
	(0.723)	(0.437)	(0.523)	(0.569)
FA	−0.0041	0.1138***	0.1067*	0.1025*
	(0.063)	(0.041)	(0.060)	(0.059)
GC	−0.0044	0.1512***	0.2913***	0.1330***
	(0.062)	(0.042)	(0.053)	(0.050)
PD		−0.0000		
		(0.000)		
DP		0.0113***		
		(0.004)		
Constant	1.0321	−2.6553***	−5.3737***	−2.7941***
	(0.930)	(0.640)	(0.799)	(0.802)
Fixed	Yes	Yes	Yes	Yes
Observations	580	580	580	472
*R* ^2^	0.7289	0.8825	0.8452	0.8317

***, **, and * represent 1, 5, and 10% levels of significance.

In Column (1), the core explanatory variable—new urbanization (NU)—has a coefficient of 1.2718, which is lower than that observed in the baseline regression but remains statistically significant at the 1% level. This confirms that the effect of NU on residential carbon emissions is stable even when key variables are substituted.

In Column (2), two additional control variables—population density (PD) and demographic profile (DP)—are introduced. The coefficient for NU remains significantly positive (8.5614), reinforcing the robustness of its effect. Notably, economic level (EL) continues to be highly significant, suggesting its consistent role in influencing carbon emissions across model specifications.

Column (3) presents the results after removing outliers. The NU coefficient increases to 9.5835, indicating that the observed effect is not driven by extreme values in the dataset. This further validates the consistency of the NU-emissions relationship.

Finally, in Column (4), the sample period is shortened, reducing the number of observations to 472. Even under this constraint, the NU coefficient remains significant (8.5749), confirming the temporal stability of the effect.

Across all models, the coefficients of other control variables—particularly EL, energy intensity (EI), and government capacity (GC)—are generally stable and statistically significant. The adjusted R^2^ values remain relatively high (ranging from 0.7289 to 0.8825), indicating good model fit and explanatory power.

These robustness tests collectively confirm the reliability and consistency of the core finding: new urbanization significantly contributes to increased carbon emissions from residential buildings, and this relationship holds under various model specifications and sample treatments.

## 5 Conclusions and recommendations

### 5.1 Conclusion

This study employs multiple econometric models to elucidate the complex mechanisms through which new urbanization affects carbon emissions from residential buildings, with three key contributions: (1) Identifying the diminishing marginal effect of ER and the U-shaped moderating pattern of STI, providing quantitative benchmarks for determining policy intervention thresholds; (2) Proposing a differentiated policy framework of “innovation-driven development in eastern regions versus regulation-prioritized approaches in central-western regions” based on geospatial heterogeneity; (3) Establishing a relatively comprehensive urbanization evaluation model that verifies STI’s critical role in breaking the “carbon lock-in” effect. Future research could incorporate emerging variables such as digital technology applications and green finance to extend the analytical dimensions.

### 5.2 Recommendations

Drawing on empirical evidence, this study formulates a coherent policy framework for low-carbon urban development in China’s new urbanization context. The integrated approach addresses three critical dimensions: First, urban planning must systematically incorporate energy-saving measures through rigorous building efficiency standards, low-carbon design promotion, and equitable resource allocation across development zones to counter residential emissions growth. Second, region-specific strategies should be implemented, with eastern cities focusing on regulatory optimization and industry-academia-research partnerships for clean technology deployment, while central/western cities prioritize infrastructure upgrades and enhanced research and development investments. Third, a city-tiered implementation system should be established, where megacities lead in adopting advanced environmental policies and green technology integration, complemented by small-medium cities implementing cost-effective solutions through periodic evaluation mechanisms and incremental innovation capacity building via regional technology hubs. This multi-scalar policy architecture ensures systematic emissions reduction while accommodating regional disparities and urban heterogeneity.

## Data Availability

The raw data supporting the conclusions of this article will be made available by the authors, without undue reservation.
