# Supplementary material for: Threshold and mediating effects of new urbanization on residential building carbon emissions: evidence from Chinese pilot cities
Source: Front Public Health. 2025 Sep 3;13:1628610. doi: 10.3389/fpubh.2025.1628610 (PMC12440927; doi:10.3389/fpubh.2025.1628610)
Supplement: Supplementary file 1 [file Table_1.docx]

| Variable | Selection notes | Measurement | Data source |
| --- | --- | --- | --- |
| IS | New urbanization policies directly reduce carbon emissions by optimizing the industrial environment, limiting the expansion of highly polluting industries and achieving industrial upgrading^32^. | Share of secondary and tertiary industries | China city Statistical Yearbook |
| EL | Accelerated urbanization at higher economic levels tends to increase carbon emissions, but also provides funds to support green technologies, thus offsetting some of the negative impacts^33^. | Logarithmic measurement of investment in fixed assets | China Statistical Yearbook |
| EI | High energy intensity means that more energy is needed to sustain current economic activity, typically increasing carbon emissions^34^. | Energy consumption per unit of GDP | China Energy Statistical Yearbook |
| FA | Increased building size increases heating, cooling, electricity demand and material consumption, leading to higher energy use and carbon emissions^35^. | Land for construction of urban residential buildings | China Urban and Rural Construction Statistical Yearbook |
| GC | Efficient governments reduce carbon emissions from buildings through policy development and promotion of energy efficiency standards, application of green technologies and energy regulation^36^. | Sum of public revenues and expenditures | Statistical bulletin on social development |

Table 1 Pilot cities for new urbanization

| Province | Cities |
| --- | --- |
| Hebei | Shijiazhuang |
| Liaoning | Dalian |
| Jilin Province | Changchun, Jilin |
| Heilongjiang Province | Harbin, Qiqihar, Mudanjiang |
| Jiangsu Province | Nanjing, Wuxi, Xuzhou, Changzhou, Suzhou, Nantong, Lianyungang, Huaian, Yancheng, Yangzhou, Zhenjiang, Taizhou, Suqian |
| Zhejiang Province | Ningbo, Jiaxing |
| Anhui Province | Hefei , Huangshan , Wuhu , Maanshan, Anqing , Huainan, Fuyang , Huaibei , Tongling , Bozhou , Xuancheng , Bengbu , Lu'an , Chuzhou , Chizhou , Suzhou , |
| Fujian Province | Putian |
| Jiangxi Province | Yingtan |
| Shandong Province | Qingdao, Dezhou, Weihai |
| Henan Province | Luoyang |
| Hubei Province | Wuhan, Xiaogan |
| Hunan Province | Changsha, Zhuzhou |
| Guangdong Province | Guangzhou, Dongguan, Huizhou |
| Guangxi Zhuang Autonomous Region | Liuzhou, Laibin |
| Sichuan Province | Luzhou |
| Guizhou Province | Anshun |
| Yunnan Province | Qujing |
| Gansu Province | Jinchang |
| Ningxia Hui Autonomous Region | Guyuan |

Table 2 Scores of new urbanization development level of pilot cities

|  | 2012 | 2013 | 2014 | 2015 | 2016 | 2017 | 2018 | 2019 | 2020 | 2021 |
| --- | --- | --- | --- | --- | --- | --- | --- | --- | --- | --- |
| Shijiazhuang | 0.2785 | 0.2714 | 0.2521 | 0.2705 | 0.2343 | 0.2349 | 0.2789 | 0.3006 | 0.3187 | 0.3510 |
| Dalian | 0.3217 | 0.2349 | 0.2789 | 0.3006 | 0.2798 | 0.3510 | 0.3217 | 0.3265 | 0.4229 | 0.3267 |
| Changchun | 0.3571 | 0.3510 | 0.3217 | 0.3265 | 0.2260 | 0.3267 | 0.2798 | 0.2896 | 0.3125 | 0.3928 |
| Jilin | 0.1834 | 0.3267 | 0.2798 | 0.2896 | 0.1889 | 0.3928 | 0.4229 | 0.4556 | 0.3274 | 0.3360 |
| Harbin | 0.3354 | 0.3928 | 0.4229 | 0.4556 | 0.2847 | 0.3360 | 0.3603 | 0.3585 | 0.4104 | 0.2154 |
| Qiqihar | 0.2477 | 0.3360 | 0.3603 | 0.3585 | 0.2365 | 0.2154 | 0.2500 | 0.2761 | 0.3092 | 0.3362 |
| Mudanjiang | 0.2908 | 0.2154 | 0.2500 | 0.2761 | 0.2480 | 0.3362 | 0.1834 | 0.2121 | 0.3441 | 0.2183 |
| Mudanjiang | 0.3186 | 0.3362 | 0.1834 | 0.2121 | 0.3255 | 0.2183 | 0.1889 | 0.2039 | 0.4516 | 0.2782 |
| Wuxi | 0.3118 | 0.2183 | 0.1889 | 0.2039 | 0.2943 | 0.2782 | 0.3274 | 0.3333 | 0.3836 | 0.3303 |
| Xuzhou | 0.2744 | 0.2782 | 0.3274 | 0.3333 | 0.2370 | 0.3303 | 0.3312 | 0.3484 | 0.3348 | 0.2901 |
| Changzhou | 0.3086 | 0.3303 | 0.3312 | 0.3484 | 0.2735 | 0.2901 | 0.3546 | 0.4146 | 0.3672 | 0.4178 |
| Suzhou | 0.3416 | 0.2901 | 0.3546 | 0.4146 | 0.3397 | 0.4178 | 0.2477 | 0.2245 | 0.4445 | 0.2431 |
| Nantong | 0.2866 | 0.4178 | 0.2477 | 0.2245 | 0.3073 | 0.2431 | 0.2365 | 0.2075 | 0.4232 | 0.2847 |
| Lianyungang | 0.1990 | 0.2431 | 0.2365 | 0.2075 | 0.2052 | 0.2847 | 0.3092 | 0.3102 | 0.3041 | 0.2718 |
| Huai'an | 0.3053 | 0.2847 | 0.3092 | 0.3102 | 0.2680 | 0.2718 | 0.2790 | 0.2934 | 0.3908 | 0.2625 |
| Yancheng | 0.1958 | 0.2718 | 0.2790 | 0.2934 | 0.1938 | 0.2625 | 0.2972 | 0.3333 | 0.2876 | 0.3245 |
| Yangzhou | 0.2678 | 0.2625 | 0.2972 | 0.3333 | 0.2521 | 0.3245 | 0.3186 | 0.3400 | 0.3727 | 0.3587 |
| Zhenjiang | 0.2572 | 0.3245 | 0.3186 | 0.3400 | 0.2516 | 0.3587 | 0.3255 | 0.2936 | 0.3500 | 0.4432 |
| Taizhou | 0.2214 | 0.3587 | 0.3255 | 0.2936 | 0.4639 | 0.4432 | 0.4516 | 0.6071 | 0.3295 | 0.3318 |
| Suqian | 0.1896 | 0.4432 | 0.4516 | 0.6071 | 0.1793 | 0.3318 | 0.3270 | 0.3303 | 0.2685 | 0.2741 |
| Ningbo | 0.3074 | 0.3318 | 0.3270 | 0.3303 | 0.2713 | 0.2741 | 0.3392 | 0.3699 | 0.3759 | 0.3894 |
| Jiaxing | 0.3098 | 0.2741 | 0.3392 | 0.3699 | 0.2738 | 0.3894 | 0.2744 | 0.2600 | 0.4010 | 0.2806 |
| Hefei | 0.2810 | 0.3894 | 0.2744 | 0.2600 | 0.2354 | 0.2806 | 0.2370 | 0.2349 | 0.3402 | 0.3261 |
| Huangshan | 0.1702 | 0.2806 | 0.2370 | 0.2349 | 0.1678 | 0.3261 | 0.3348 | 0.3447 | 0.2763 | 0.3110 |
| Wuhu | 0.1982 | 0.3261 | 0.3348 | 0.3447 | 0.1991 | 0.3110 | 0.3066 | 0.3047 | 0.2807 | 0.2685 |
| Maanshan | 0.2683 | 0.3110 | 0.3066 | 0.3047 | 0.2311 | 0.2685 | 0.3286 | 0.3633 | 0.3385 | 0.3796 |
| Anqing | 0.1593 | 0.2685 | 0.3286 | 0.3633 | 0.1823 | 0.3796 | 0.3416 | 0.3694 | 0.2845 | 0.3626 |
| Huainan | 0.1735 | 0.3796 | 0.3416 | 0.3694 | 0.1448 | 0.3626 | 0.3397 | 0.3366 | 0.2353 | 0.4479 |
| Fuyang | 0.1263 | 0.3626 | 0.3397 | 0.3366 | 0.1540 | 0.4479 | 0.4445 | 0.4475 | 0.2487 | 0.2862 |
| Huaibei | 0.2122 | 0.4479 | 0.4445 | 0.4475 | 0.1828 | 0.2862 | 0.3259 | 0.3460 | 0.2922 | 0.3058 |
| Tongling | 0.2254 | 0.2862 | 0.3259 | 0.3460 | 0.1980 | 0.3058 | 0.3816 | 0.4176 | 0.3501 | 0.4322 |
| Huizhou | 0.2289 | 0.3058 | 0.3816 | 0.4176 | 0.2238 | 0.4322 | 0.1990 | 0.2000 | 0.3542 | 0.2441 |
| Xuancheng | 0.1942 | 0.4322 | 0.1990 | 0.2000 | 0.2025 | 0.2441 | 0.2052 | 0.2103 | 0.3199 | 0.2892 |
| Bengbu | 0.2011 | 0.2441 | 0.2052 | 0.2103 | 0.1902 | 0.2892 | 0.3041 | 0.3198 | 0.3112 | 0.2830 |
| Lu'an | 0.1542 | 0.2892 | 0.3041 | 0.3198 | 0.1749 | 0.2830 | 0.3122 | 0.3231 | 0.2744 | 0.2736 |
| Chuzhou | 0.1818 | 0.2830 | 0.3122 | 0.3231 | 0.1827 | 0.2736 | 0.3369 | 0.3736 | 0.2923 | 0.3740 |
| Chizhou | 0.1454 | 0.2736 | 0.3369 | 0.3736 | 0.1528 | 0.3740 | 0.1958 | 0.1978 | 0.2434 | 0.2257 |
| Suzhou | 0.1518 | 0.3740 | 0.1958 | 0.1978 | 0.1831 | 0.2257 | 0.1938 | 0.1904 | 0.2795 | 0.2735 |
| Putian | 0.2015 | 0.2257 | 0.1938 | 0.1904 | 0.1935 | 0.2735 | 0.2876 | 0.3054 | 0.2707 | 0.2591 |
| Yingtan | 0.2136 | 0.2735 | 0.2876 | 0.3054 | 0.1984 | 0.2591 | 0.2740 | 0.2908 | 0.2996 | 0.2464 |
| Qingdao | 0.2739 | 0.2591 | 0.2740 | 0.2908 | 0.2724 | 0.2464 | 0.3248 | 0.3573 | 0.3723 | 0.3793 |
| Weihai | 0.3444 | 0.2464 | 0.3248 | 0.3573 | 0.2904 | 0.3793 | 0.2572 | 0.2607 | 0.3676 | 0.2886 |
| Dezhou | 0.2033 | 0.3793 | 0.2572 | 0.2607 | 0.1995 | 0.2886 | 0.2516 | 0.2388 | 0.2753 | 0.3196 |
| Luoyang | 0.2309 | 0.2886 | 0.2516 | 0.2388 | 0.2083 | 0.3196 | 0.3500 | 0.3358 | 0.3125 | 0.2875 |
| Wuhan | 0.2687 | 0.3196 | 0.3500 | 0.3358 | 0.2820 | 0.2875 | 0.3213 | 0.2576 | 0.4576 | 0.2229 |
| Xiaogan | 0.1765 | 0.2875 | 0.3213 | 0.2576 | 0.1733 | 0.2229 | 0.2811 | 0.3123 | 0.2650 | 0.3478 |
| Changsha | 0.2630 | 0.2229 | 0.2811 | 0.3123 | 0.2474 | 0.3478 | 0.1896 | 0.1726 | 0.3510 | 0.2156 |
| Zhuzhou | 0.1850 | 0.3478 | 0.1896 | 0.1726 | 0.1789 | 0.2156 | 0.1793 | 0.1751 | 0.2731 | 0.2558 |
| Guangzhou | 0.4171 | 0.2156 | 0.1793 | 0.1751 | 0.3610 | 0.2558 | 0.2685 | 0.3004 | 0.4911 | 0.3156 |
| Dongguan | 0.3715 | 0.2558 | 0.2685 | 0.3004 | 0.3412 | 0.3156 | 0.3282 | 0.3173 | 0.4434 | 0.2554 |
| Huizhou | 0.2457 | 0.3156 | 0.3282 | 0.3173 | 0.2102 | 0.2554 | 0.3191 | 0.3577 | 0.2888 | 0.3929 |
| Liuzhou | 0.2124 | 0.2554 | 0.3191 | 0.3577 | 0.1921 | 0.3929 | 0.3098 | 0.3199 | 0.3096 | 0.3149 |
| Laibin | 0.1664 | 0.3929 | 0.3098 | 0.3199 | 0.1635 | 0.3149 | 0.2738 | 0.2616 | 0.2586 | 0.3760 |
| Luzhou | 0.1582 | 0.3760 | 0.4010 | 0.4236 | 0.1646 | 0.2597 | 0.2771 | 0.2681 | 0.2792 | 0.2330 |
| Anshun | 0.1226 | 0.2597 | 0.2771 | 0.2681 | 0.1808 | 0.2330 | 0.2956 | 0.3383 | 0.2510 | 0.3417 |
| Qujing | 0.2437 | 0.2330 | 0.2956 | 0.3383 | 0.2128 | 0.3417 | 0.1702 | 0.2060 | 0.3027 | 0.1915 |
| Jinchang | 0.3092 | 0.3417 | 0.1702 | 0.2060 | 0.2644 | 0.1915 | 0.1678 | 0.1712 | 0.3692 | 0.2460 |
| Guyuan | 0.2719 | 0.1915 | 0.1678 | 0.1712 | 0.2112 | 0.2460 | 0.2763 | 0.3034 | 0.3811 | 0.2011 |

Table 2 Pilot City Population Urbanization Score

|  | 2012 | 2013 | 2014 | 2015 | 2016 | 2017 | 2018 | 2019 | 2020 | 2021 |
| --- | --- | --- | --- | --- | --- | --- | --- | --- | --- | --- |
| Shijiazhuang | 0.3408 | 0.3850 | 0.4114 | 0.4169 | 0.4641 | 0.5012 | 0.4916 | 0.5134 | 0.5297 | 0.5640 |
| Dalian | 0.6328 | 0.6684 | 0.6691 | 0.6131 | 0.6689 | 0.7366 | 0.7005 | 0.7084 | 0.6854 | 0.6920 |
| Changchun | 0.3902 | 0.4484 | 0.4478 | 0.4446 | 0.4921 | 0.5276 | 0.5097 | 0.6075 | 0.5684 | 0.5760 |
| Jilin | 0.4093 | 0.4474 | 0.4452 | 0.4390 | 0.4766 | 0.4992 | 0.4833 | 0.4749 | 0.5108 | 0.4868 |
| Harbin | 0.3897 | 0.3832 | 0.3032 | 0.4246 | 0.4770 | 0.5142 | 0.4740 | 0.5129 | 0.5809 | 0.5780 |
| Qiqihar | 0.2732 | 0.2738 | 0.2440 | 0.2979 | 0.2469 | 0.2634 | 0.2844 | 0.2894 | 0.4142 | 0.3833 |
| Mudanjiang | 0.4080 | 0.4494 | 0.4346 | 0.4049 | 0.4448 | 0.4633 | 0.4006 | 0.4883 | 0.5640 | 0.5145 |
| Mudanjiang | 0.6533 | 0.6954 | 0.7157 | 0.7635 | 0.7926 | 0.8153 | 0.8002 | 0.8108 | 0.8555 | 0.9943 |
| Wuxi | 0.6428 | 0.6976 | 0.7256 | 0.7240 | 0.7427 | 0.7552 | 0.7486 | 0.7586 | 0.8065 | 0.7410 |
| Xuzhou | 0.5476 | 0.5799 | 0.6161 | 0.6369 | 0.6369 | 0.6474 | 0.6590 | 0.6729 | 0.6665 | 0.6420 |
| Changzhou | 0.6067 | 0.6641 | 0.6876 | 0.7016 | 0.7150 | 0.7302 | 0.7262 | 0.7345 | 0.7695 | 0.7817 |
| Suzhou | 0.5980 | 0.6888 | 0.7158 | 0.7155 | 0.7318 | 0.7485 | 0.7429 | 0.7504 | 0.7887 | 0.7882 |
| Nantong | 0.5292 | 0.5779 | 0.6131 | 0.6386 | 0.6492 | 0.6644 | 0.6732 | 0.6833 | 0.7091 | 0.6771 |
| Lianyungang | 0.5317 | 0.5550 | 0.5606 | 0.6239 | 0.6325 | 0.6509 | 0.6571 | 0.6622 | 0.6498 | 0.6570 |
| Huai'an | 0.5460 | 0.5546 | 0.5688 | 0.5888 | 0.6272 | 0.6406 | 0.6503 | 0.6600 | 0.6825 | 0.5020 |
| Yancheng | 0.4863 | 0.5161 | 0.5632 | 0.6077 | 0.6091 | 0.6180 | 0.6367 | 0.6420 | 0.5804 | 0.5694 |
| Yangzhou | 0.5543 | 0.5795 | 0.6099 | 0.6328 | 0.6517 | 0.6709 | 0.6799 | 0.6907 | 0.7153 | 0.6110 |
| Zhenjiang | 0.6020 | 0.6215 | 0.6732 | 0.6843 | 0.6962 | 0.7116 | 0.7116 | 0.7217 | 0.7855 | 0.7185 |
| Taizhou | 0.5035 | 0.5637 | 0.5969 | 0.6241 | 0.6298 | 0.6471 | 0.6601 | 0.6710 | 0.6874 | 0.6905 |
| Suqian | 0.4522 | 0.4778 | 0.5104 | 0.5632 | 0.5673 | 0.5728 | 0.5969 | 0.6124 | 0.6186 | 0.6363 |
| Ningbo | 0.5994 | 0.6610 | 0.6885 | 0.6826 | 0.6967 | 0.7092 | 0.7196 | 0.7650 | 0.7291 | 0.7198 |
| Jiaxing | 0.4513 | 0.4822 | 0.5000 | 0.5053 | 0.5349 | 0.5726 | 0.5628 | 0.6897 | 0.7042 | 0.7010 |
| Hefei | 0.4557 | 0.5421 | 0.5869 | 0.5912 | 0.6105 | 0.6582 | 0.6273 | 0.6855 | 0.7032 | 0.7299 |
| Huangshan | 0.3180 | 0.3650 | 0.3775 | 0.3712 | 0.4011 | 0.4368 | 0.4865 | 0.5390 | 0.5561 | 0.5664 |
| Wuhu | 0.3766 | 0.4646 | 0.4766 | 0.4850 | 0.5153 | 0.5757 | 0.5758 | 0.6444 | 0.6797 | 0.7109 |
| Maanshan | 0.5284 | 0.5532 | 0.5650 | 0.5450 | 0.5745 | 0.5973 | 0.6087 | 0.6535 | 0.6438 | 0.6693 |
| Anqing | 0.1964 | 0.2305 | 0.2881 | 0.3951 | 0.3832 | 0.4477 | 0.4672 | 0.5288 | 0.5374 | 0.6872 |
| Huainan | 0.4031 | 0.4600 | 0.4681 | 0.3772 | 0.4329 | 0.5404 | 0.5203 | 0.4851 | 0.5386 | 0.5498 |
| Fuyang | 0.3109 | 0.3861 | 0.4742 | 0.4250 | 0.3688 | 0.4394 | 0.4483 | 0.4380 | 0.4159 | 0.4240 |
| Huaibei | 0.3331 | 0.3933 | 0.4074 | 0.3740 | 0.4265 | 0.5953 | 0.5899 | 0.5796 | 0.5921 | 0.5972 |
| Tongling | 0.5298 | 0.6288 | 0.6141 | 0.4167 | 0.4875 | 0.5289 | 0.5185 | 0.5922 | 0.6286 | 0.6339 |
| Huizhou | 0.1514 | 0.1715 | 0.2142 | 0.2065 | 0.3204 | 0.3248 | 0.3529 | 0.3607 | 0.3941 | 0.3882 |
| Xuancheng | 0.3398 | 0.3776 | 0.3867 | 0.4217 | 0.4359 | 0.4593 | 0.4517 | 0.5181 | 0.5461 | 0.6006 |
| Bengbu | 0.4114 | 0.4391 | 0.4441 | 0.4485 | 0.5020 | 0.5008 | 0.4821 | 0.5447 | 0.5405 | 0.5600 |
| Lu'an | 0.2250 | 0.2515 | 0.2770 | 0.3353 | 0.3611 | 0.3826 | 0.3998 | 0.4359 | 0.4659 | 0.4755 |
| Chuzhou | 0.2920 | 0.3508 | 0.3830 | 0.3861 | 0.4114 | 0.4457 | 0.4688 | 0.5277 | 0.5440 | 0.5753 |
| Chizhou | 0.3196 | 0.3579 | 0.3797 | 0.4659 | 0.4274 | 0.4604 | 0.4916 | 0.5869 | 0.5184 | 0.5894 |
| Suzhou | 0.2138 | 0.2416 | 0.2884 | 0.3387 | 0.3846 | 0.3369 | 0.3901 | 0.4088 | 0.3731 | 0.5917 |
| Putian | 0.5387 | 0.5418 | 0.5587 | 0.5803 | 0.5853 | 0.5663 | 0.5959 | 0.6333 | 0.5481 | 0.5629 |
| Yingtan | 0.3792 | 0.4078 | 0.4210 | 0.4772 | 0.5020 | 0.5258 | 0.5230 | 0.5411 | 0.5548 | 0.5602 |
| Qingdao | 0.5442 | 0.5746 | 0.5828 | 0.5670 | 0.5965 | 0.6332 | 0.6245 | 0.6310 | 0.6711 | 0.6917 |
| Weihai | 0.6854 | 0.6763 | 0.6954 | 0.7194 | 0.7119 | 0.7106 | 0.6975 | 0.7053 | 0.6748 | 0.6886 |
| Dezhou | 0.4057 | 0.4136 | 0.4523 | 0.4742 | 0.5010 | 0.5274 | 0.5363 | 0.5159 | 0.5137 | 0.5495 |
| Luoyang | 0.4827 | 0.4876 | 0.5067 | 0.5275 | 0.5108 | 0.5356 | 0.4150 | 0.4569 | 0.5171 | 0.4961 |
| Wuhan | 0.4420 | 0.5194 | 0.5769 | 0.5612 | 0.6659 | 0.7035 | 0.7436 | 0.7623 | 0.7608 | 0.7352 |
| Xiaogan | 0.1543 | 0.1632 | 0.2155 | 0.2852 | 0.3603 | 0.3435 | 0.3779 | 0.4115 | 0.3723 | 0.4397 |
| Changsha | 0.5596 | 0.5930 | 0.6088 | 0.6255 | 0.6531 | 0.6925 | 0.6950 | 0.6848 | 0.6792 | 0.7513 |
| Zhuzhou | 0.4856 | 0.4094 | 0.5321 | 0.5462 | 0.5117 | 0.5507 | 0.6339 | 0.6295 | 0.6587 | 0.7193 |
| Guangzhou | 0.7119 | 0.7760 | 0.7650 | 0.7366 | 0.7530 | 0.7756 | 0.7981 | 0.7761 | 0.7833 | 0.8020 |
| Dongguan | 0.7480 | 0.8002 | 0.7903 | 0.7552 | 0.8032 | 0.8292 | 0.8857 | 0.9127 | 0.8596 | 0.9051 |
| Huizhou | 0.6042 | 0.6371 | 0.6368 | 0.6089 | 0.6219 | 0.6362 | 0.6387 | 0.6537 | 0.6900 | 0.6859 |
| Liuzhou | 0.3699 | 0.4189 | 0.4341 | 0.4053 | 0.4624 | 0.6180 | 0.5743 | 0.5489 | 0.5753 | 0.6074 |
| Laibin | 0.2757 | 0.2820 | 0.3023 | 0.3520 | 0.3348 | 0.4248 | 0.3952 | 0.3759 | 0.4417 | 0.4299 |
| Luzhou | 0.3722 | 0.3703 | 0.3841 | 0.3522 | 0.3705 | 0.4206 | 0.4493 | 0.5155 | 0.4931 | 0.5045 |
| Anshun | 0.2920 | 0.3121 | 0.3304 | 0.4172 | 0.4167 | 0.4260 | 0.4182 | 0.4407 | 0.3390 | 0.2975 |
| Qujing | 0.1947 | 0.2584 | 0.2768 | 0.3178 | 0.3652 | 0.3685 | 0.4557 | 0.4464 | 0.3059 | 0.2942 |
| Jinchang | 0.5216 | 0.5704 | 0.5893 | 0.5524 | 0.6011 | 0.6410 | 0.6142 | 0.6439 | 0.6709 | 0.6859 |
| Guyuan | 0.1784 | 0.2172 | 0.2501 | 0.2758 | 0.2913 | 0.2946 | 0.3003 | 0.3194 | 0.4018 | 0.3222 |

Table 3 Pilot City Economic Urbanization Score

|  | 2012 | 2013 | 2014 | 2015 | 2016 | 2017 | 2018 | 2019 | 2020 | 2021 |
| --- | --- | --- | --- | --- | --- | --- | --- | --- | --- | --- |
| Shijiazhuang | 0.0731 | 0.0898 | 0.0912 | 0.1081 | 0.0846 | 0.0985 | 0.1222 | 0.1786 | 0.1578 | 0.1712 |
| Dalian | 0.1814 | 0.2309 | 0.1904 | 0.2111 | 0.1347 | 0.1354 | 0.1945 | 0.2750 | 0.2339 | 0.2596 |
| Changchun | 0.1066 | 0.1271 | 0.1288 | 0.1469 | 0.1056 | 0.1135 | 0.1637 | 0.2260 | 0.1963 | 0.2080 |
| Jilin | 0.0922 | 0.1032 | 0.0955 | 0.1027 | 0.0713 | 0.0935 | 0.1080 | 0.1286 | 0.1076 | 0.1255 |
| Harbin | 0.0866 | 0.1001 | 0.1012 | 0.1294 | 0.0975 | 0.1121 | 0.1377 | 0.1908 | 0.1605 | 0.1656 |
| Qiqihar | 0.0328 | 0.0387 | 0.0482 | 0.0600 | 0.0478 | 0.0690 | 0.0729 | 0.1043 | 0.0882 | 0.1021 |
| Mudanjiang | 0.0519 | 0.0758 | 0.0753 | 0.0929 | 0.0732 | 0.1193 | 0.1005 | 0.1228 | 0.1025 | 0.1124 |
| Mudanjiang | 0.1868 | 0.2448 | 0.2192 | 0.2449 | 0.1886 | 0.1854 | 0.2782 | 0.4395 | 0.3869 | 0.4268 |
| Wuxi | 0.2113 | 0.3014 | 0.2244 | 0.2528 | 0.1897 | 0.1667 | 0.2538 | 0.3827 | 0.3645 | 0.4135 |
| Xuzhou | 0.0859 | 0.0866 | 0.1016 | 0.1193 | 0.0851 | 0.1005 | 0.1366 | 0.2103 | 0.1903 | 0.2171 |
| Changzhou | 0.1652 | 0.2404 | 0.1961 | 0.2205 | 0.1706 | 0.1603 | 0.2496 | 0.3742 | 0.3339 | 0.3768 |
| Suzhou | 0.2095 | 0.3436 | 0.2320 | 0.2597 | 0.1998 | 0.1783 | 0.2870 | 0.4146 | 0.3592 | 0.4076 |
| Nantong | 0.1200 | 0.1388 | 0.1523 | 0.1730 | 0.1321 | 0.1297 | 0.1955 | 0.2974 | 0.2776 | 0.3101 |
| Lianyungang | 0.0740 | 0.0739 | 0.0887 | 0.1039 | 0.0779 | 0.0900 | 0.1203 | 0.2030 | 0.1824 | 0.2069 |
| Huai'an | 0.0744 | 0.0787 | 0.0973 | 0.1158 | 0.0879 | 0.0989 | 0.1345 | 0.2074 | 0.1987 | 0.2280 |
| Yancheng | 0.0743 | 0.0798 | 0.0946 | 0.1144 | 0.0871 | 0.0952 | 0.1360 | 0.2205 | 0.2076 | 0.2376 |
| Yangzhou | 0.1213 | 0.1381 | 0.1460 | 0.1724 | 0.1258 | 0.1271 | 0.1939 | 0.2856 | 0.2713 | 0.3065 |
| Zhenjiang | 0.1256 | 0.1526 | 0.1766 | 0.1870 | 0.1417 | 0.1355 | 0.2037 | 0.2848 | 0.2723 | 0.2901 |
| Taizhou | 0.1027 | 0.1126 | 0.1307 | 0.1529 | 0.7445 | 0.1157 | 0.1710 | 0.2591 | 0.2499 | 0.3060 |
| Suqian | 0.0479 | 0.0559 | 0.0724 | 0.0885 | 0.0634 | 0.0760 | 0.1011 | 0.1698 | 0.1555 | 0.1776 |
| Ningbo | 0.1708 | 0.2373 | 0.1968 | 0.2170 | 0.1749 | 0.1523 | 0.2435 | 0.3736 | 0.3186 | 0.3714 |
| Jiaxing | 0.1258 | 0.1763 | 0.1517 | 0.1726 | 0.1465 | 0.1271 | 0.2005 | 0.3061 | 0.2688 | 0.3026 |
| Hefei | 0.1100 | 0.1300 | 0.1306 | 0.1534 | 0.1151 | 0.1201 | 0.1799 | 0.2959 | 0.2620 | 0.2924 |
| Huangshan | 0.0569 | 0.0641 | 0.0751 | 0.0935 | 0.0719 | 0.0948 | 0.1141 | 0.1821 | 0.1674 | 0.1909 |
| Wuhu | 0.0920 | 0.0975 | 0.1146 | 0.1312 | 0.0986 | 0.1002 | 0.1505 | 0.2351 | 0.2237 | 0.2601 |
| Maanshan | 0.1133 | 0.1214 | 0.1221 | 0.1312 | 0.1076 | 0.1004 | 0.1587 | 0.2408 | 0.2381 | 0.2754 |
| Anqing | 0.0441 | 0.0428 | 0.0555 | 0.0700 | 0.0569 | 0.0654 | 0.0893 | 0.1552 | 0.1509 | 0.1719 |
| Huainan | 0.0857 | 0.0825 | 0.0896 | 0.0806 | 0.0608 | 0.0726 | 0.0954 | 0.1753 | 0.1525 | 0.1721 |
| Fuyang | 0.0232 | 0.0232 | 0.0372 | 0.0494 | 0.0450 | 0.0556 | 0.0670 | 0.1345 | 0.1160 | 0.1269 |
| Huaibei | 0.0782 | 0.0716 | 0.0751 | 0.0773 | 0.0563 | 0.0696 | 0.0954 | 0.1657 | 0.1567 | 0.1716 |
| Tongling | 0.1447 | 0.1573 | 0.1528 | 0.1175 | 0.0865 | 0.0866 | 0.1342 | 0.1788 | 0.1861 | 0.2173 |
| Huizhou | 0.1169 | 0.0958 | 0.0815 | 0.0712 | 0.0447 | 0.0478 | 0.0433 | 0.0657 | 0.0409 | 0.0334 |
| Xuancheng | 0.0637 | 0.0755 | 0.0815 | 0.0926 | 0.0775 | 0.0796 | 0.1079 | 0.1803 | 0.1663 | 0.1883 |
| Bengbu | 0.0485 | 0.0533 | 0.0665 | 0.0830 | 0.0686 | 0.0770 | 0.1053 | 0.1773 | 0.1610 | 0.1664 |
| Lu'an | 0.0261 | 0.0301 | 0.0410 | 0.0587 | 0.0514 | 0.0630 | 0.0810 | 0.1449 | 0.1313 | 0.1366 |
| Chuzhou | 0.0483 | 0.0510 | 0.0652 | 0.0788 | 0.0629 | 0.0668 | 0.0945 | 0.1887 | 0.1773 | 0.2005 |
| Chizhou | 0.0516 | 0.0562 | 0.0687 | 0.0803 | 0.0632 | 0.0763 | 0.1004 | 0.1765 | 0.1647 | 0.1912 |
| Suzhou | 0.0338 | 0.0285 | 0.0395 | 0.0546 | 0.0473 | 0.0645 | 0.0761 | 0.1268 | 0.1170 | 0.1275 |
| Putian | 0.0739 | 0.0783 | 0.0976 | 0.1107 | 0.0875 | 0.0869 | 0.1325 | 0.2068 | 0.1827 | 0.2045 |
| Yingtan | 0.0598 | 0.0703 | 0.0873 | 0.1196 | 0.0797 | 0.0822 | 0.1261 | 0.1990 | 0.1852 | 0.2158 |
| Qingdao | 0.1515 | 0.1950 | 0.1794 | 0.2057 | 0.1552 | 0.1545 | 0.2265 | 0.3292 | 0.3029 | 0.3404 |
| Weihai | 0.1414 | 0.1700 | 0.1577 | 0.1829 | 0.1355 | 0.1330 | 0.2000 | 0.2378 | 0.2243 | 0.2563 |
| Dezhou | 0.0580 | 0.0733 | 0.0801 | 0.0881 | 0.0617 | 0.0779 | 0.1002 | 0.1533 | 0.1371 | 0.1527 |
| Luoyang | 0.0793 | 0.0792 | 0.0868 | 0.0898 | 0.0790 | 0.0928 | 0.1259 | 0.1952 | 0.1713 | 0.1870 |
| Wuhan | 0.1426 | 0.1971 | 0.1695 | 0.1984 | 0.1454 | 0.1510 | 0.2273 | 0.3454 | 0.3062 | 0.3252 |
| Xiaogan | 0.0204 | 0.0312 | 0.0435 | 0.0546 | 0.0508 | 0.0512 | 0.0723 | 0.1145 | 0.1316 | 0.1548 |
| Changsha | 0.1597 | 0.1990 | 0.1875 | 0.2156 | 0.1634 | 0.1570 | 0.2364 | 0.3411 | 0.2915 | 0.3168 |
| Zhuzhou | 0.0831 | 0.0943 | 0.1054 | 0.1231 | 0.0988 | 0.0913 | 0.1381 | 0.2151 | 0.1972 | 0.2170 |
| Guangzhou | 0.2209 | 0.3462 | 0.2418 | 0.2742 | 0.2001 | 0.2037 | 0.2876 | 0.4199 | 0.3546 | 0.3912 |
| Dongguan | 0.1471 | 0.4600 | 0.1283 | 0.1495 | 0.1241 | 0.1200 | 0.1771 | 0.2566 | 0.2176 | 0.2481 |
| Huizhou | 0.0924 | 0.1428 | 0.1106 | 0.1281 | 0.1017 | 0.1037 | 0.1532 | 0.2298 | 0.1866 | 0.2155 |
| Liuzhou | 0.0754 | 0.0977 | 0.1028 | 0.1145 | 0.0730 | 0.0889 | 0.1332 | 0.2013 | 0.1852 | 0.1909 |
| Laibin | 0.0539 | 0.0426 | 0.0544 | 0.0573 | 0.0624 | 0.0656 | 0.0825 | 0.1140 | 0.1027 | 0.1267 |
| Luzhou | 0.0370 | 0.0457 | 0.0620 | 0.0732 | 0.0630 | 0.0622 | 0.0921 | 0.1619 | 0.1300 | 0.1610 |
| Anshun | 0.0355 | 0.0406 | 0.0587 | 0.0862 | 0.0631 | 0.0835 | 0.0953 | 0.1586 | 0.1278 | 0.1443 |
| Qujing | 0.0407 | 0.0465 | 0.0524 | 0.0610 | 0.0592 | 0.0638 | 0.0887 | 0.1610 | 0.1536 | 0.1704 |
| Jinchang | 0.1028 | 0.0981 | 0.0974 | 0.0986 | 0.0750 | 0.0758 | 0.1120 | 0.1992 | 0.1907 | 0.2173 |
| Guyuan | 0.0420 | 0.0359 | 0.0465 | 0.0759 | 0.0563 | 0.6969 | 0.0878 | 0.1397 | 0.1437 | 0.1506 |

Table 4 Pilot city social urbanization score

|  | 2012 | 2013 | 2014 | 2015 | 2016 | 2017 | 2018 | 2019 | 2020 | 2021 |
| --- | --- | --- | --- | --- | --- | --- | --- | --- | --- | --- |
| Shijiazhuang | 0.2884 | 0.3135 | 0.2496 | 0.2599 | 0.2615 | 0.2646 | 0.2876 | 0.3054 | 0.2737 | 0.2903 |
| Dalian | 0.3332 | 0.3678 | 0.3721 | 0.3935 | 0.4126 | 0.4616 | 0.4916 | 0.4981 | 0.5546 | 0.6402 |
| Changchun | 0.3618 | 0.3471 | 0.3455 | 0.3290 | 0.2127 | 0.1831 | 0.1643 | 0.1699 | 0.2796 | 0.3115 |
| Jilin | 0.1990 | 0.2633 | 0.2666 | 0.2141 | 0.2215 | 0.2741 | 0.2940 | 0.3216 | 0.3886 | 0.4069 |
| Harbin | 0.3241 | 0.3942 | 0.3692 | 0.3937 | 0.3655 | 0.3814 | 0.4197 | 0.5123 | 0.4189 | 0.4552 |
| Qiqihar | 0.2307 | 0.2526 | 0.2553 | 0.2235 | 0.4134 | 0.2536 | 0.2773 | 0.3048 | 0.2941 | 0.2688 |
| Mudanjiang | 0.3629 | 0.3702 | 0.3360 | 0.3858 | 0.4005 | 0.4095 | 0.4188 | 0.4285 | 0.3893 | 0.3835 |
| Mudanjiang | 0.2682 | 0.3090 | 0.2383 | 0.2998 | 0.3304 | 0.2201 | 0.2273 | 0.3852 | 0.3744 | 0.4024 |
| Wuxi | 0.2579 | 0.2896 | 0.3091 | 0.2986 | 0.3256 | 0.3020 | 0.3217 | 0.3378 | 0.3427 | 0.3551 |
| Xuzhou | 0.1925 | 0.2126 | 0.2122 | 0.2241 | 0.2382 | 0.2528 | 0.2638 | 0.2752 | 0.2674 | 0.2762 |
| Changzhou | 0.2487 | 0.2468 | 0.2581 | 0.2375 | 0.2574 | 0.2863 | 0.3049 | 0.3341 | 0.3249 | 0.3363 |
| Suzhou | 0.3385 | 0.3299 | 0.3529 | 0.3555 | 0.4361 | 0.4750 | 0.4957 | 0.5077 | 0.4805 | 0.4824 |
| Nantong | 0.2786 | 0.2917 | 0.3338 | 0.3528 | 0.3962 | 0.4265 | 0.4398 | 0.4471 | 0.4255 | 0.4367 |
| Lianyungang | 0.1421 | 0.1901 | 0.1819 | 0.1824 | 0.1735 | 0.2129 | 0.2190 | 0.2381 | 0.2597 | 0.2645 |
| Huai'an | 0.3467 | 0.3469 | 0.3399 | 0.3608 | 0.3609 | 0.3807 | 0.4063 | 0.4239 | 0.4192 | 0.4057 |
| Yancheng | 0.1481 | 0.1915 | 0.1969 | 0.1758 | 0.1774 | 0.1750 | 0.1927 | 0.2080 | 0.2429 | 0.2593 |
| Yangzhou | 0.2514 | 0.2757 | 0.2681 | 0.2733 | 0.2788 | 0.2848 | 0.3331 | 0.3583 | 0.3562 | 0.3747 |
| Zhenjiang | 0.2292 | 0.2750 | 0.2975 | 0.2539 | 0.2549 | 0.2557 | 0.2679 | 0.2749 | 0.3135 | 0.2555 |
| Taizhou | 0.1882 | 0.4613 | 0.5024 | 0.2393 | 0.2387 | 0.2380 | 0.2555 | 0.2744 | 0.2800 | 0.2907 |
| Suqian | 0.1111 | 0.1335 | 0.1375 | 0.1395 | 0.1450 | 0.1540 | 0.1628 | 0.2027 | 0.1937 | 0.2270 |
| Ningbo | 0.2949 | 0.3124 | 0.3438 | 0.3157 | 0.3150 | 0.3124 | 0.3184 | 0.3397 | 0.3671 | 0.3881 |
| Jiaxing | 0.4262 | 0.4816 | 0.4745 | 0.4188 | 0.4147 | 0.3943 | 0.4361 | 0.4095 | 0.4499 | 0.5187 |
| Hefei | 0.1786 | 0.1933 | 0.1892 | 0.1699 | 0.1811 | 0.1846 | 0.2078 | 0.2406 | 0.2257 | 0.2288 |
| Huangshan | 0.1679 | 0.3253 | 0.1995 | 0.2008 | 0.2031 | 0.1971 | 0.2237 | 0.2190 | 0.2603 | 0.2762 |
| Wuhu | 0.1380 | 0.1586 | 0.1960 | 0.1633 | 0.1938 | 0.1926 | 0.2055 | 0.1940 | 0.1900 | 0.2151 |
| Maanshan | 0.2115 | 0.2279 | 0.2275 | 0.2182 | 0.2266 | 0.2290 | 0.2591 | 0.2678 | 0.2950 | 0.3402 |
| Anqing | 0.1603 | 0.2243 | 0.1602 | 0.1691 | 0.2751 | 0.1517 | 0.1921 | 0.1916 | 0.2377 | 0.3227 |
| Huainan | 0.1023 | 0.1190 | 0.1286 | 0.0969 | 0.1112 | 0.0941 | 0.1205 | 0.1454 | 0.1730 | 0.1867 |
| Fuyang | 0.0875 | 0.1123 | 0.1035 | 0.1020 | 0.1145 | 0.1231 | 0.1429 | 0.1709 | 0.1757 | 0.1988 |
| Huaibei | 0.1902 | 0.2068 | 0.2073 | 0.1920 | 0.1934 | 0.1950 | 0.1966 | 0.2164 | 0.2414 | 0.2480 |
| Tongling | 0.1830 | 0.2308 | 0.3411 | 0.1576 | 0.2042 | 0.2677 | 0.2831 | 0.2536 | 0.4057 | 0.3973 |
| Huizhou | 0.1808 | 0.1881 | 0.2047 | 0.2402 | 0.2681 | 0.3613 | 0.3981 | 0.4067 | 0.3912 | 0.4352 |
| Xuancheng | 0.1679 | 0.1955 | 0.2001 | 0.1906 | 0.2243 | 0.2490 | 0.2549 | 0.2856 | 0.3034 | 0.3158 |
| Bengbu | 0.1681 | 0.1875 | 0.1861 | 0.1682 | 0.1863 | 0.1874 | 0.1961 | 0.2177 | 0.3089 | 0.3224 |
| Lu'an | 0.1113 | 0.1454 | 0.1401 | 0.1660 | 0.1692 | 0.1835 | 0.1992 | 0.2129 | 0.2239 | 0.2623 |
| Chuzhou | 0.1122 | 0.1348 | 0.1375 | 0.1252 | 0.1496 | 0.1837 | 0.1867 | 0.1903 | 0.2062 | 0.2373 |
| Chizhou | 0.0914 | 0.1355 | 0.1144 | 0.1266 | 0.1233 | 0.1385 | 0.1507 | 0.1506 | 0.1840 | 0.2189 |
| Suzhou | 0.1434 | 0.1768 | 0.1800 | 0.1938 | 0.2040 | 0.2349 | 0.2456 | 0.2528 | 0.2594 | 0.2725 |
| Putian | 0.1571 | 0.1683 | 0.1580 | 0.1818 | 0.1719 | 0.1758 | 0.1868 | 0.1888 | 0.1916 | 0.2018 |
| Yingtan | 0.1765 | 0.2010 | 0.1943 | 0.1885 | 0.1843 | 0.1804 | 0.2059 | 0.2094 | 0.2381 | 0.2535 |
| Qingdao | 0.2278 | 0.2886 | 0.3015 | 0.2768 | 0.2811 | 0.2786 | 0.2802 | 0.2969 | 0.2812 | 0.3017 |
| Weihai | 0.2607 | 0.3419 | 0.4831 | 0.3049 | 0.3149 | 0.2676 | 0.2790 | 0.3011 | 0.3182 | 0.3622 |
| Dezhou | 0.1278 | 0.1825 | 0.1641 | 0.1484 | 0.1447 | 0.1391 | 0.1453 | 0.1822 | 0.2035 | 0.2175 |
| Luoyang | 0.1663 | 0.1989 | 0.1931 | 0.1917 | 0.1921 | 0.1877 | 0.1926 | 0.2091 | 0.2141 | 0.2588 |
| Wuhan | 0.1934 | 0.3154 | 0.3025 | 0.3525 | 0.3524 | 0.3512 | 0.3658 | 0.3713 | 0.3850 | 0.3842 |
| Xiaogan | 0.1872 | 0.2235 | 0.2196 | 0.2469 | 0.2192 | 0.2288 | 0.2504 | 0.2619 | 0.2741 | 0.2585 |
| Changsha | 0.2292 | 0.2663 | 0.2556 | 0.2493 | 0.2650 | 0.2153 | 0.2200 | 0.2759 | 0.3262 | 0.3402 |
| Zhuzhou | 0.1282 | 0.1519 | 0.1443 | 0.1477 | 0.1738 | 0.1616 | 0.2174 | 0.2327 | 0.2182 | 0.3671 |
| Guangzhou | 0.3841 | 0.3954 | 0.4888 | 0.3779 | 0.3943 | 0.4105 | 0.4584 | 0.4679 | 0.4643 | 0.4632 |
| Dongguan | 0.3161 | 0.3145 | 0.3756 | 0.2974 | 0.2869 | 0.2893 | 0.2521 | 0.2595 | 0.2515 | 0.2826 |
| Huizhou | 0.2249 | 0.2323 | 0.2377 | 0.1892 | 0.1866 | 0.1855 | 0.1619 | 0.1728 | 0.1858 | 0.2087 |
| Liuzhou | 0.1931 | 0.2132 | 0.2111 | 0.2102 | 0.2085 | 0.2275 | 0.2259 | 0.2412 | 0.2607 | 0.2803 |
| Laibin | 0.1233 | 0.1788 | 0.1439 | 0.1628 | 0.1777 | 0.1605 | 0.1794 | 0.2136 | 0.2270 | 0.2406 |
| Luzhou | 0.1618 | 0.2607 | 0.2696 | 0.2160 | 0.2223 | 0.2221 | 0.2264 | 0.2418 | 0.2808 | 0.2808 |
| Anshun | 0.1248 | 0.1439 | 0.1314 | 0.1388 | 0.1493 | 0.1572 | 0.1571 | 0.1661 | 0.1935 | 0.3073 |
| Qujing | 0.1909 | 0.1758 | 0.1897 | 0.1838 | 0.2138 | 0.1960 | 0.3099 | 0.3088 | 0.2933 | 0.2983 |
| Jinchang | 0.2365 | 0.2339 | 0.2367 | 0.2737 | 0.2771 | 0.2820 | 0.2927 | 0.3329 | 0.3104 | 0.5174 |
| Guyuan | 0.3203 | 0.3406 | 0.3160 | 0.3169 | 0.3166 | 0.3061 | 0.3143 | 0.3628 | 0.3726 | 0.4351 |

Table 5 Pilot City Spatial Urbanization Score

|  | 2012 | 2013 | 2014 | 2015 | 2016 | 2017 | 2018 | 2019 | 2020 | 2021 |
| --- | --- | --- | --- | --- | --- | --- | --- | --- | --- | --- |
| Shijiazhuang | 0.3180 | 0.2903 | 0.2450 | 0.2586 | 0.2587 | 0.2569 | 0.2093 | 0.1924 | 0.2365 | 0.2719 |
| Dalian | 0.2083 | 0.2051 | 0.2016 | 0.2045 | 0.2017 | 0.1911 | 0.2007 | 0.2087 | 0.2166 | 0.2383 |
| Changchun | 0.4717 | 0.4314 | 0.4417 | 0.4568 | 0.2507 | 0.2386 | 0.2035 | 0.2108 | 0.2125 | 0.2266 |
| Jilin | 0.0675 | 0.1278 | 0.1354 | 0.1569 | 0.1595 | 0.1566 | 0.1572 | 0.1572 | 0.1934 | 0.1882 |
| Harbin | 0.4505 | 0.4138 | 0.4203 | 0.4228 | 0.4093 | 0.3930 | 0.4002 | 0.4003 | 0.4226 | 0.3947 |
| Qiqihar | 0.3816 | 0.3253 | 0.3304 | 0.3446 | 0.3357 | 0.3175 | 0.3117 | 0.3025 | 0.3119 | 0.2720 |
| Mudanjiang | 0.3454 | 0.2927 | 0.2937 | 0.3029 | 0.2953 | 0.2825 | 0.2751 | 0.2688 | 0.2683 | 0.2068 |
| Mudanjiang | 0.2310 | 0.2714 | 0.2792 | 0.2838 | 0.3011 | 0.3117 | 0.3175 | 0.3295 | 0.3449 | 0.6981 |
| Wuxi | 0.1923 | 0.2000 | 0.2046 | 0.2052 | 0.1992 | 0.2115 | 0.2094 | 0.2087 | 0.2052 | 0.2119 |
| Xuzhou | 0.2891 | 0.2736 | 0.2908 | 0.2346 | 0.2328 | 0.2321 | 0.2347 | 0.2405 | 0.2471 | 0.2363 |
| Changzhou | 0.2452 | 0.2385 | 0.2217 | 0.2119 | 0.2135 | 0.2205 | 0.1976 | 0.2024 | 0.1978 | 0.1939 |
| Suzhou | 0.2495 | 0.2581 | 0.2635 | 0.2705 | 0.2880 | 0.2956 | 0.2884 | 0.2809 | 0.2708 | 0.2639 |
| Nantong | 0.2186 | 0.2373 | 0.2657 | 0.2886 | 0.2911 | 0.2964 | 0.2909 | 0.2949 | 0.3065 | 0.3024 |
| Lianyungang | 0.1289 | 0.1384 | 0.1486 | 0.1828 | 0.1872 | 0.1990 | 0.1813 | 0.1828 | 0.1818 | 0.1878 |
| Huai'an | 0.2835 | 0.2691 | 0.2923 | 0.2934 | 0.2694 | 0.2840 | 0.2599 | 0.2672 | 0.2779 | 0.2690 |
| Yancheng | 0.1420 | 0.1381 | 0.1256 | 0.1348 | 0.1401 | 0.1513 | 0.1611 | 0.1583 | 0.1548 | 0.1637 |
| Yangzhou | 0.1545 | 0.1550 | 0.1635 | 0.1726 | 0.1732 | 0.1877 | 0.1955 | 0.2013 | 0.2086 | 0.2152 |
| Zhenjiang | 0.1236 | 0.1365 | 0.1460 | 0.1518 | 0.1507 | 0.1595 | 0.1610 | 0.1631 | 0.1674 | 0.1721 |
| Taizhou | 0.1264 | 0.1433 | 0.1496 | 0.1646 | 0.1630 | 0.1794 | 0.1698 | 0.1833 | 0.1917 | 0.1951 |
| Suqian | 0.1679 | 0.1320 | 0.1407 | 0.1478 | 0.1379 | 0.1423 | 0.1366 | 0.1368 | 0.1397 | 0.1634 |
| Ningbo | 0.2168 | 0.2147 | 0.2265 | 0.2169 | 0.1822 | 0.1824 | 0.1820 | 0.1825 | 0.2115 | 0.2159 |
| Jiaxing | 0.1983 | 0.1880 | 0.1952 | 0.2063 | 0.1988 | 0.2001 | 0.2074 | 0.2230 | 0.2298 | 0.2242 |
| Hefei | 0.3296 | 0.2625 | 0.2691 | 0.2432 | 0.2545 | 0.2589 | 0.2661 | 0.2734 | 0.2783 | 0.2413 |
| Huangshan | 0.0563 | 0.0669 | 0.0777 | 0.0802 | 0.0802 | 0.0897 | 0.0956 | 0.0993 | 0.1070 | 0.1333 |
| Wuhu | 0.1399 | 0.1523 | 0.1524 | 0.1526 | 0.1560 | 0.1573 | 0.1519 | 0.1491 | 0.1626 | 0.1725 |
| Maanshan | 0.2065 | 0.1934 | 0.1990 | 0.2107 | 0.2050 | 0.2080 | 0.2068 | 0.2063 | 0.2130 | 0.2237 |
| Anqing | 0.1072 | 0.1075 | 0.1165 | 0.1163 | 0.1255 | 0.1199 | 0.1475 | 0.1783 | 0.2012 | 0.2422 |
| Huainan | 0.0938 | 0.1002 | 0.0898 | 0.0967 | 0.0971 | 0.0997 | 0.0984 | 0.1002 | 0.1144 | 0.1203 |
| Fuyang | 0.1228 | 0.1350 | 0.1651 | 0.1795 | 0.1821 | 0.1880 | 0.1901 | 0.1801 | 0.1939 | 0.1936 |
| Huaibei | 0.1408 | 0.1300 | 0.1355 | 0.1509 | 0.1542 | 0.1574 | 0.1583 | 0.1628 | 0.1657 | 0.1597 |
| Tongling | 0.0943 | 0.1209 | 0.1230 | 0.1376 | 0.1307 | 0.1346 | 0.1269 | 0.1254 | 0.1641 | 0.1290 |
| Huizhou | 0.2683 | 0.2743 | 0.3095 | 0.3488 | 0.3478 | 0.3302 | 0.3330 | 0.3325 | 0.3476 | 0.3275 |
| Xuancheng | 0.1725 | 0.1673 | 0.1793 | 0.1969 | 0.1965 | 0.2148 | 0.2173 | 0.2179 | 0.2204 | 0.2217 |
| Bengbu | 0.1719 | 0.1400 | 0.1587 | 0.1763 | 0.1774 | 0.1806 | 0.1841 | 0.1970 | 0.1981 | 0.1946 |
| Lu'an | 0.1732 | 0.1681 | 0.1791 | 0.1962 | 0.1926 | 0.1986 | 0.1978 | 0.1967 | 0.1977 | 0.2000 |
| Chuzhou | 0.1861 | 0.2071 | 0.2376 | 0.2498 | 0.2148 | 0.2175 | 0.2154 | 0.2032 | 0.2119 | 0.2250 |
| Chizhou | 0.0816 | 0.0863 | 0.0924 | 0.0945 | 0.0868 | 0.0952 | 0.0913 | 0.0930 | 0.0894 | 0.0997 |
| Suzhou | 0.1862 | 0.1826 | 0.2027 | 0.2125 | 0.2128 | 0.2341 | 0.2355 | 0.2338 | 0.2344 | 0.2242 |
| Putian | 0.0818 | 0.0788 | 0.0845 | 0.1402 | 0.1499 | 0.1470 | 0.1520 | 0.1488 | 0.1622 | 0.1663 |
| Yingtan | 0.1817 | 0.1627 | 0.1661 | 0.1760 | 0.1789 | 0.1716 | 0.1629 | 0.1530 | 0.2070 | 0.2027 |
| Qingdao | 0.1893 | 0.1633 | 0.2415 | 0.2502 | 0.2284 | 0.2459 | 0.2543 | 0.2798 | 0.2787 | 0.3054 |
| Weihai | 0.3050 | 0.2923 | 0.2093 | 0.2201 | 0.2093 | 0.2181 | 0.2158 | 0.2096 | 0.2223 | 0.2257 |
| Dezhou | 0.1486 | 0.1622 | 0.1823 | 0.1880 | 0.1808 | 0.1709 | 0.1651 | 0.1722 | 0.1772 | 0.1720 |
| Luoyang | 0.2233 | 0.2077 | 0.2149 | 0.2387 | 0.2459 | 0.2382 | 0.2522 | 0.2815 | 0.2911 | 0.2728 |
| Wuhan | 0.2632 | 0.3811 | 0.3853 | 0.2472 | 0.2565 | 0.3551 | 0.3916 | 0.4121 | 0.4393 | 0.1402 |
| Xiaogan | 0.1664 | 0.1508 | 0.1861 | 0.2232 | 0.1907 | 0.1893 | 0.1903 | 0.1835 | 0.1826 | 0.1709 |
| Changsha | 0.1559 | 0.1571 | 0.1606 | 0.1470 | 0.1640 | 0.1605 | 0.1805 | 0.1799 | 0.2175 | 0.2049 |
| Zhuzhou | 0.0741 | 0.0842 | 0.0931 | 0.0989 | 0.0945 | 0.1119 | 0.1083 | 0.1083 | 0.1183 | 0.2426 |
| Guangzhou | 0.4157 | 0.3999 | 0.3939 | 0.3383 | 0.3509 | 0.3635 | 0.3857 | 0.3823 | 0.3861 | 0.3857 |
| Dongguan | 0.4036 | 0.4222 | 0.4312 | 0.4365 | 0.4482 | 0.4468 | 0.4576 | 0.4916 | 0.4915 | 0.5188 |
| Huizhou | 0.1309 | 0.1308 | 0.1375 | 0.1326 | 0.1325 | 0.1336 | 0.1376 | 0.1608 | 0.1788 | 0.1789 |
| Liuzhou | 0.1540 | 0.1513 | 0.1564 | 0.1630 | 0.1675 | 0.1896 | 0.2034 | 0.2112 | 0.2377 | 0.2378 |
| Laibin | 0.1447 | 0.1455 | 0.1687 | 0.1795 | 0.1765 | 0.1911 | 0.2043 | 0.2048 | 0.2079 | 0.1960 |
| Luzhou | 0.0904 | 0.0948 | 0.1004 | 0.1092 | 0.1201 | 0.1345 | 0.1477 | 0.1497 | 0.1641 | 0.1566 |
| Anshun | 0.0979 | 0.0959 | 0.1126 | 0.1548 | 0.1706 | 0.1753 | 0.1649 | 0.1716 | 0.1704 | 0.2158 |
| Qujing | 0.3563 | 0.3202 | 0.3400 | 0.3292 | 0.3470 | 0.2765 | 0.2818 | 0.2768 | 0.2850 | 0.2595 |
| Jinchang | 0.3822 | 0.3374 | 0.3504 | 0.3110 | 0.3021 | 0.2994 | 0.3001 | 0.2820 | 0.2917 | 0.3788 |
| Guyuan | 0.3980 | 0.3681 | 0.3724 | 0.3821 | 0.2732 | 0.2666 | 0.2999 | 0.2927 | 0.3179 | 0.2858 |

Table 6 Pilot city eco-urbanization score

|  | 2012 | 2013 | 2014 | 2015 | 2016 | 2017 | 2018 | 2019 | 2020 | 2021 |
| --- | --- | --- | --- | --- | --- | --- | --- | --- | --- | --- |
| Shijiazhuang | 0.5458 | 0.5822 | 0.5849 | 0.6508 | 0.6675 | 0.6973 | 0.6936 | 0.7126 | 0.7212 | 0.7266 |
| Dalian | 0.5598 | 0.5772 | 0.5806 | 0.6066 | 0.6081 | 0.6386 | 0.6506 | 0.6441 | 0.6720 | 0.6796 |
| Changchun | 0.5028 | 0.5147 | 0.5877 | 0.5991 | 0.5722 | 0.6161 | 0.6218 | 0.6213 | 0.6362 | 0.6500 |
| Jilin | 0.4811 | 0.5145 | 0.5107 | 0.5545 | 0.5678 | 0.5866 | 0.6171 | 0.6742 | 0.6809 | 0.6884 |
| Harbin | 0.4626 | 0.4854 | 0.4753 | 0.4775 | 0.4828 | 0.5061 | 0.5110 | 0.5268 | 0.5440 | 0.5456 |
| Qiqihar | 0.3578 | 0.3534 | 0.3612 | 0.3882 | 0.4474 | 0.4803 | 0.5071 | 0.5298 | 0.5567 | 0.6857 |
| Mudanjiang | 0.2904 | 0.3002 | 0.3552 | 0.3775 | 0.3797 | 0.3890 | 0.4358 | 0.5247 | 0.5779 | 0.5911 |
| Mudanjiang | 0.6170 | 0.6250 | 0.6391 | 0.6648 | 0.6714 | 0.6765 | 0.6774 | 0.6845 | 0.6957 | 0.7000 |
| Wuxi | 0.6437 | 0.6479 | 0.6524 | 0.6551 | 0.6556 | 0.6578 | 0.6588 | 0.6624 | 0.6655 | 0.6670 |
| Xuzhou | 0.5832 | 0.6249 | 0.6328 | 0.6533 | 0.6622 | 0.6632 | 0.6650 | 0.6654 | 0.6809 | 0.7008 |
| Changzhou | 0.6039 | 0.6160 | 0.6241 | 0.6355 | 0.6473 | 0.6555 | 0.6609 | 0.6654 | 0.6668 | 0.6808 |
| Suzhou | 0.5932 | 0.6067 | 0.6060 | 0.6198 | 0.6264 | 0.6328 | 0.6407 | 0.6493 | 0.6588 | 0.6621 |
| Nantong | 0.5892 | 0.6232 | 0.6651 | 0.6719 | 0.6992 | 0.7115 | 0.7164 | 0.7313 | 0.7043 | 0.7177 |
| Lianyungang | 0.4944 | 0.5312 | 0.5669 | 0.5902 | 0.5985 | 0.6024 | 0.6127 | 0.6340 | 0.6470 | 0.6613 |
| Huai'an | 0.4973 | 0.5253 | 0.5790 | 0.5858 | 0.6227 | 0.6353 | 0.6302 | 0.6389 | 0.6400 | 0.6639 |
| Yancheng | 0.4548 | 0.5082 | 0.5686 | 0.5823 | 0.5917 | 0.6151 | 0.6288 | 0.6366 | 0.6581 | 0.6762 |
| Yangzhou | 0.6769 | 0.6788 | 0.6912 | 0.7009 | 0.7057 | 0.7108 | 0.7176 | 0.7258 | 0.7359 | 0.7431 |
| Zhenjiang | 0.6561 | 0.6777 | 0.6920 | 0.6997 | 0.7039 | 0.7083 | 0.7128 | 0.6972 | 0.7018 | 0.7080 |
| Taizhou | 0.5117 | 0.5212 | 0.5222 | 0.5427 | 0.5678 | 0.6380 | 0.6461 | 0.6547 | 0.6645 | 0.6779 |
| Suqian | 0.5025 | 0.5416 | 0.5683 | 0.6457 | 0.6519 | 0.6558 | 0.6629 | 0.6679 | 0.6984 | 0.7053 |
| Ningbo | 0.5347 | 0.5421 | 0.5487 | 0.5614 | 0.5756 | 0.5788 | 0.6120 | 0.6400 | 0.6447 | 0.6669 |
| Jiaxing | 0.5782 | 0.5777 | 0.5817 | 0.5881 | 0.5780 | 0.6160 | 0.6586 | 0.6623 | 0.6746 | 0.6787 |
| Hefei | 0.5488 | 0.5690 | 0.6182 | 0.6169 | 0.6082 | 0.6330 | 0.6280 | 0.6262 | 0.6246 | 0.6350 |
| Huangshan | 0.6443 | 0.6714 | 0.6769 | 0.6768 | 0.6813 | 0.6774 | 0.6882 | 0.6941 | 0.7206 | 0.7530 |
| Wuhu | 0.5621 | 0.5777 | 0.5604 | 0.5572 | 0.6040 | 0.6084 | 0.6088 | 0.6186 | 0.6066 | 0.6588 |
| Maanshan | 0.6622 | 0.6742 | 0.6713 | 0.6606 | 0.6733 | 0.6675 | 0.6714 | 0.6738 | 0.6902 | 0.6877 |
| Anqing | 0.5085 | 0.5544 | 0.5750 | 0.5768 | 0.6107 | 0.6264 | 0.6459 | 0.6629 | 0.6294 | 0.6661 |
| Huainan | 0.5373 | 0.5570 | 0.5823 | 0.5820 | 0.5902 | 0.6545 | 0.6482 | 0.6426 | 0.6193 | 0.6255 |
| Fuyang | 0.2996 | 0.4174 | 0.4294 | 0.4423 | 0.5395 | 0.5541 | 0.6994 | 0.6826 | 0.6677 | 0.7069 |
| Huaibei | 0.6594 | 0.6690 | 0.6712 | 0.6768 | 0.6995 | 0.6948 | 0.7179 | 0.7249 | 0.7258 | 0.7389 |
| Tongling | 0.5692 | 0.6316 | 0.6583 | 0.6457 | 0.7235 | 0.7354 | 0.7032 | 0.6060 | 0.7172 | 0.7182 |
| Huizhou | 0.5453 | 0.5605 | 0.5453 | 0.5883 | 0.5732 | 0.5641 | 0.6107 | 0.6345 | 0.6760 | 0.7052 |
| Xuancheng | 0.4626 | 0.5917 | 0.5682 | 0.6245 | 0.6238 | 0.6373 | 0.6449 | 0.6259 | 0.6761 | 0.7197 |
| Bengbu | 0.4575 | 0.5045 | 0.5375 | 0.6009 | 0.6122 | 0.6244 | 0.6300 | 0.6392 | 0.6550 | 0.6643 |
| Lu'an | 0.4778 | 0.5373 | 0.5550 | 0.6042 | 0.6255 | 0.6317 | 0.6414 | 0.6453 | 0.6768 | 0.6810 |
| Chuzhou | 0.5445 | 0.5791 | 0.5580 | 0.5869 | 0.5905 | 0.6217 | 0.6441 | 0.7446 | 0.7140 | 0.7384 |
| Chizhou | 0.5658 | 0.5814 | 0.6656 | 0.6525 | 0.6607 | 0.6862 | 0.6917 | 0.7040 | 0.7354 | 0.7481 |
| Suzhou | 0.3184 | 0.4975 | 0.5064 | 0.5668 | 0.5659 | 0.6230 | 0.6107 | 0.5974 | 0.6266 | 0.6972 |
| Putian | 0.5895 | 0.5992 | 0.5920 | 0.5842 | 0.5934 | 0.6324 | 0.6539 | 0.6679 | 0.6887 | 0.6924 |
| Yingtan | 0.5520 | 0.5907 | 0.5981 | 0.6765 | 0.6501 | 0.8118 | 0.6442 | 0.6385 | 0.6768 | 0.7120 |
| Qingdao | 0.6051 | 0.6114 | 0.6259 | 0.6126 | 0.6935 | 0.6775 | 0.6835 | 0.6978 | 0.7325 | 0.7181 |
| Weihai | 0.8177 | 0.8183 | 0.8197 | 0.8340 | 0.8367 | 0.8390 | 0.8418 | 0.8567 | 0.8561 | 0.8645 |
| Dezhou | 0.7650 | 0.7741 | 0.7905 | 0.8010 | 0.7933 | 0.7538 | 0.7372 | 0.7282 | 0.7586 | 0.8133 |
| Luoyang | 0.4244 | 0.4545 | 0.5149 | 0.5149 | 0.5613 | 0.5921 | 0.5855 | 0.5679 | 0.6686 | 0.6885 |
| Wuhan | 0.5187 | 0.5415 | 0.5581 | 0.5689 | 0.5604 | 0.5498 | 0.5512 | 0.5586 | 0.6424 | 0.6687 |
| Xiaogan | 0.5655 | 0.5961 | 0.4764 | 0.4655 | 0.4953 | 0.5016 | 0.5368 | 0.5338 | 0.5822 | 0.6740 |
| Changsha | 0.5098 | 0.5362 | 0.5615 | 0.5606 | 0.5634 | 0.5418 | 0.5975 | 0.5967 | 0.6014 | 0.6410 |
| Zhuzhou | 0.5354 | 0.5652 | 0.5527 | 0.5789 | 0.6043 | 0.6301 | 0.6384 | 0.6531 | 0.6416 | 0.6256 |
| Guangzhou | 0.6332 | 0.6694 | 0.6561 | 0.7170 | 0.7269 | 0.7499 | 0.7747 | 0.7994 | 0.7975 | 0.8013 |
| Dongguan | 0.5372 | 0.5990 | 0.6025 | 0.6602 | 0.7744 | 0.7855 | 0.7958 | 0.7641 | 0.7537 | 0.7841 |
| Huizhou | 0.5727 | 0.6246 | 0.6394 | 0.6622 | 0.6950 | 0.7028 | 0.6839 | 0.6805 | 0.6815 | 0.6618 |
| Liuzhou | 0.5752 | 0.5913 | 0.5927 | 0.6243 | 0.6282 | 0.6351 | 0.6548 | 0.6417 | 0.5852 | 0.6412 |
| Laibin | 0.4608 | 0.4789 | 0.4553 | 0.4791 | 0.4841 | 0.4841 | 0.4758 | 0.5638 | 0.5602 | 0.5810 |
| Luzhou | 0.4041 | 0.4585 | 0.4401 | 0.5050 | 0.5361 | 0.5763 | 0.5984 | 0.6253 | 0.6348 | 0.6395 |
| Anshun | 0.1333 | 0.1920 | 0.1756 | 0.4515 | 0.6568 | 0.6497 | 0.6649 | 0.6658 | 0.7636 | 0.8455 |
| Qujing | 0.5136 | 0.4971 | 0.5288 | 0.5162 | 0.4750 | 0.2940 | 0.5656 | 0.6022 | 0.6061 | 0.6200 |
| Jinchang | 0.4849 | 0.5535 | 0.6674 | 0.6886 | 0.7163 | 0.7494 | 0.7973 | 0.7638 | 0.7481 | 0.8359 |
| Guyuan | 0.3409 | 0.3127 | 0.3195 | 0.3588 | 0.5097 | 0.6710 | 0.7107 | 0.7797 | 0.9311 | 0.9126 |
